# Drought hazards and stakeholder perception: Unraveling the interlinkages between drought severity, perceived impacts, preparedness, and management

**DOI:** 10.1007/s13280-023-01849-w

**Published:** 2023-04-03

**Authors:** Claudia Teutschbein, Frederike Albrecht, Malgorzata Blicharska, Faranak Tootoonchi, Elin Stenfors, Thomas Grabs

**Affiliations:** 1 Air, Water and Landscape Science, Department of Earth Sciences, Uppsala University, Villavägen 16, 75236 Uppsala, Sweden; 2 Department of Political Science and Law, Swedish Defence University, Box 278 05, 11593 Stockholm, Sweden; 3 Natural Resources and Sustainable Development, Department of Earth Sciences, Uppsala University, Villavägen 16, 75236 Uppsala, Sweden; 4 Centre of Natural Hazards and Disaster Science (CNDS), Villavägen 16, 75236 Uppsala, Sweden

**Keywords:** Drought, Stakeholder perception, Municipal drought planning, Drought preparedness, Drought risk management, Climate change adaptation

## Abstract

The future risk for droughts and water shortages calls for substantial efforts by authorities to adapt at local levels. Understanding their perception of drought hazards, risk and vulnerability can help to identify drivers of and barriers to drought risk planning and management in a changing climate at the local level. This paper presents a novel interdisciplinary drought case study in Sweden that integrates soft data from a nationwide survey among more than 100 local practitioners and hard data based on hydrological measurements to provide a holistic assessment of the links between drought severity and the perceived levels of drought severity, impacts, preparedness, and management for two consecutive drought events. The paper highlights challenges for drought risk planning and management in a changing climate at the local level and elaborates on how improved understanding of local practitioners to plan for climate change adaptation can be achieved.

**Supplementary Information:**

The online version contains supplementary material available at 10.1007/s13280-023-01849-w.

## Introduction

Droughts are a natural part of climate: they always have and will always take place. They are multifaceted natural hazards caused by a lack of precipitation (i.e., drier than normal conditions) that can occur in any region of the world and are not restricted to dry climate zones (Wilhite 1996; WMO and GWP 2016). In contrast to other natural hazards like floods or earthquakes, drought events develop slowly and are cumulative in nature (Şen 2015), which means they become increasingly severe over time given an insufficient water input to the system (Zaidman et al. 2002). They are sometimes even called ‘creeping disasters’ (Van Loon 2015), as they are one of the most costly natural hazards affecting many societal sectors.

Drought risk depends on the combination of physical factors (hazard), exposure of the society and its vulnerability (IPCC 2021), where vulnerability includes susceptibility to impacts, coping capacity, and adaptive capacity (UNDRR 2021). Adequate drought risk management strategies increase a society’s capacity to cope more efficiently (Wilhite 2019), but also require measuring and addressing aspects related to the hazard, exposure, and vulnerability (Buurman et al. 2017). However, the disaster literature has to date mostly neglected droughts (Raikes et al. 2019). Simultaneously, the intensity of droughts has already increased due to climate change in recent decades (Schlaepfer et al. 2017) and the number of drought disasters has grown considerably over the years, primarily due to increasing exposure and vulnerability rather than due to an increase in frequency (UNDRR 2019; Wilhite 1996). Developing countries are typically most vulnerable to drought due to structural features that quickly turn droughts into cascading events. However, even in the global north, vulnerability to droughts is substantial and socially co-produced, e.g., by dependency chains in the society, or by the lack of drought risk management. There is thus a need for improved risk management strategies to address drought hazards and to adapt to a changing climate also in the global north (AghaKouchak et al. 2015a).

Adaptation to climate change implies the “adjustment to actual or expected climate and its effects” and aims to alleviate or even prevent negative impacts and to take advantages of potential positive consequences (IPCC 2014a). While national adaptation policies have started to be implemented (Dannevig et al. 2012), the local variation of climate change impacts require different types of local strategies, with regional and local actors responsible (Measham et al. 2011; IPCC 2014b). At the same time, only little is known about how these regional and local actors respond to an increased drought risk, what capacity they have to deal with it, what improvements are needed (Measham et al. 2011; Nordgren et al. 2016), and, particularly, what their drought related risk perceptions are that impact the design of risk planning and management approaches (Steg and Sievers 2000; Fuchs et al. 2017; Ridolfi et al. 2020). For example, it has been shown that the perceptions of climatic variability or hazards by different actors may diverge from what the actual “hard” data shows (Agrawal et al. 2020; Dakurah 2021; Salam et al. 2021), but such studies are rare. Scholars have also explored theoretically how different risk perceptions can impact risk management strategies (Ridolfi et al. 2020), but empirical applications are lacking. In our study, we address the above knowledge gaps by investigating Swedish municipalities’ perception of drought risk, by exploring challenges for drought risk planning and management, and by comparing the drought perceptions to actual “hard” data on droughts in Sweden.

Although Sweden has historically been rich in water, it is not exempt of droughts. Especially the 1976 Northwest European drought and the 2003 European heatwave will long be remembered for their devastating effects (Fink et al. 2004; Bradford 2000). More recently, late 2015 was the onset of another longer dry period (especially in southern and central Sweden), which continued to worsen in 2016 and 2017 due to a lack of precipitation. Many municipalities in the southern part of the country, where streamflow was on average 43% below normal, issued local water use restrictions in the first half of 2017 (Geological Survey of Sweden 2017). Toward the end of 2017, precipitation amounts returned to normal levels, but 2018 developed into yet another dry and unusually warm year (Swedish Government 2018a), leading to the most serious wildfires in modern history (The Local 2018). These latest drought events led to water shortages across Sweden that severely affected the environment and society, ranging from lost harvests and emergency slaughter of livestock (Sveriges Radio 2018), to lower revenues from tourism (Vattenmyndigheterna 2018), damage to aquatic ecosystems (HaV 2018), and forest fires (Swedish Government 2018a). The wildfires alone caused financial damage of over 70 million € (Vikström 2018), while the agricultural sector suffered financial damage of almost 1 billion € (Rapp 2018), ten times more than the most costly flooding damage that occurred in recent years (Elfström 2015; Nyhetsbyrån 2018). Another major societal function affected by these drought events was the drinking water supply (Vattenmyndigheterna 2018). In the future, meteorological shifts will further aggravate present-day water stress during summer, especially in southern Sweden (Eklund et al. 2015; Teutschbein et al. 2022b). Due to additional pressures such as growing population, tourism, and overexploitation of water resources, water resources management may face major challenges, which further increases the need for developing local and regional adaptation plans.

The European Commission encourages its member states to develop national disaster risk assessments (European Commission 2013) and provides a set of recommendations and guidelines for that (Casajus Valles et al. 2019), including a chapter on droughts, stating that “every Member State should have a drought management plan to cope with possible impacts” (Casajus Valles et al. 2019). This has, to our best knowledge, not yet been implemented in Sweden. The Swedish government has only recently published a Government Bill with its first strategic national plan for climate adaptation (Swedish Government 2018b). The strategy addresses a number of climate change effects in Sweden, but the issue of a reduced water availability is only briefly mentioned in relation to southern Sweden. However, considering the challenges with drinking water availability linked to recent droughts, increased attention is imperative, particularly at municipal levels where vulnerability typically unfolds (Dannevig et al. 2012). In 2010, only 12% of Swedish drinking water producers specifically considered potential effects of droughts on drinking water in their risk assessment (Norén, researcher in drinking water risk management, pers.comm. 2015). More recently, a 2017 online survey in central Sweden showed that only 27% of surveyed municipalities had an action plan for water shortages prior to 2017 (Lundkvist and Andersson 2018).

There is a lack of knowledge on how drought hazards and their associated impacts are perceived, assessed, and managed by practitioners, and if these perceptions reflect the objective risks. Therefore, the aim of this study is twofold. First, we examine the perceptions of Swedish municipalities on their local water resources, future risks of droughts, as well as their perceptions of the 2017 and 2018 droughts, including severity, impacts, local preparedness, and management of these events. Second, we compare the perceived severity of the 2017 and 2018 drought hazard with the actual severity of these droughts assessed using hydrological drought indices based on precipitation (as a proxy for surface water) and groundwater measurements. Based on the results, we discuss challenges for drought risk planning and management in a changing climate and how improved understanding of local practitioners to plan for climate change adaptation can be achieved. The study is carried out utilizing an interdisciplinary approach that combines perspectives and methods from social sciences and hydrology.

## Materials and methods

### Study area Sweden

The study was conducted in Sweden, a country in northern Europe with a total population of 10.3 million and a land area of approximately 408 000 km^2^. Sweden is a heavily forested country and, under normal conditions, one of the 50 water-richest countries in the world (FAO 2020). Sweden is subdivided into 290 municipalities organized within 21 counties. While the counties are the top-level administrative and political subdivisions, municipalities are the local government bodies. The water governance system largely relies on municipal self-government, which is important in the development of drought management strategies and has legal obligation to create local action plans. The Swedish Civil Contingencies Agency (MSB) offers support and guidance for municipalities to fulfill this task. Municipalities act as operators who are directly responsible for local water management and the implementation of necessary measures to protect local surface and groundwater bodies that are used (or maybe used in the future) for drinking water supply. It should be stressed that Sweden has implemented the principle of responsibility/accountability (‘Ansvarsprincipen’), which implies that those responsible for water management in ordinary times will also be responsible in times of crisis, such as water shortages (Swedish Defence University 2019). The responsibilities of municipalities also include a large part of all public services and infrastructure planning, such as drinking water distribution and sewerage systems (Carlsson-Kanyama 2013). For a detailed description of Sweden’s geographic/hydroclimatic features and its water governance, we refer to the Supplementary Information (Sects. S1. Study area Sweden and S2. Water management in Sweden).

### Data collection and analysis

#### Soft data—drought risk, planning, and perception

An online survey using a web-based Swedish questionnaire was sent to all 290 Swedish municipalities in December 2018 (response rate 41% with 118 unique responses). The survey combined both open- and closed-end questions (Vicente and Reis 2010), including a mixture of multiple-choice, binary, rating scale, ranking, free-text answers, and respondent-specific (e.g., name of county and municipality, job role) questions organized in six key sections: background, concepts and terminology, collaborations and action plans, summer 2018, summer 2017, and adaptation. A more detailed description of the survey design can be found in the Supplementary Information (see S3. Survey design, Fig. S1).

Responses were first analyzed by means of descriptive statistics to present the municipalities’ water management, perception of drought and flood hazard, as well as the respondents’ perception of the 2017 and 2018 droughts using Microsoft Excel and MATLAB software. These simple, descriptive statistics included summary statistics, non-parametric Spearman’s rank correlation (Spearman 1904) and crosstabulations with significance tests.

For a more in-depth analysis, the municipalities were further categorized into northern (above 60°N) and southern (below 60°N) municipalities that defer in their climate conditions and population characteristics, as well as into urban (population density > 300 per km^2^) and rural (population density < 300 per km^2^).

To unravel the link between drought management and the perceived drought risk, we computed the non-parametric Spearman’s rank correlation coefficient *ρ*_s_ (Spearman 1904) between perceived severity, impacts, preparedness, and management. Spearman rank correlation was chosen over linear Pearson product-moment correlation (Pearson 1920), because Spearman assumes only monotony without making prior assumptions about the nature of the relationships (e.g., linear or logarithmic). We further hypothesized that municipalities with action plans differ from those without plans regarding (1) the perception of severity, impacts, preparedness, and level of management, (2) the number of affected sectors, and (3) the implementation of different countermeasures when hit by a drought. We also tested whether municipalities without action plans are more likely to implement one soon if they (1) assume an increasing risk of future droughts or if they (2) recently perceived strong drought impacts.

The Wilcoxon rank sum test (Asadzadeh et al. 2014) was utilized to compare different levels of perception (i.e., interval data) between the different groups of municipalities (i.e., with vs. without drought action plans). To compare categorical responses (e.g., “yes/no”) between different groups of municipalities, we applied Fisher’s exact test (Fisher 1922).

#### Hard data—hydroclimatic analysis

To compare the perceived drought severity with the actual drought hazards, the droughts of 2017 and 2018 were also analyzed from a hydrological perspective, i.e., including assessments of (1) precipitation deficits (as a simplified proxy for surface water deficits), (2) groundwater deficits, and (3) concurrence of both precipitation deficit and extreme temperatures across the country. A brief description is provided below, for a detailed explanation on the underlying data and applied methods, we refer the reader to the Supplementary Information (see S4. Hydroclimatic Data and Analysis Methods).

Precipitation deficits were assessed using the Standardized Precipitation Index (SPI) originally developed by McKee et al. (1993). The SPI provides a dimensionless anomaly from normal situations, where positive SPI values indicate conditions above normal (i.e., wet conditions) and negative values below normal (i.e., drought conditions). The SPI was computed based on a 6-month moving average (i.e., the so-called SPI6) as it integrates precipitation anomalies over a sufficiently long period to reflect anomalies in both precipitation and surface water (e.g., streams and lakes). In addition, groundwater deficits were evaluated based on the Standardized Groundwater Index (SGI), which was developed by Bloomfield and Marchant (2013) and is based on the same concept as the SPI. All hydrological data was obtained from the Swedish Meteorological and Hydrological Institute (SMHI) and the Swedish Geological Survey (SGU), details can be found in the Supplementary Information (see S4. Hydroclimatic Data and Analysis Methods). The severity of the precipitation and groundwater deficits was assessed with a classification scheme ranging from normal conditions through mild, moderate, and severe, to extreme droughts (for details see Supplementary Information, Table S1). Based on the computed SPI6 and SGI6 values, the spatial extent and severity of the 2017 and 2018 drought were mapped and compared to the perceived severity obtained from the online survey.

We also evaluated the 2017 and 2018 droughts based on their empirical return periods, a concept commonly applied to identify critical events (e.g., floods or droughts) and provide critical information for practitioners (Hosking and Wallis 1993; Salvadori et al. 2011). We here computed both univariate and multivariate empirical return periods for observed March-August precipitation deficits and annual summer temperature anomalies over the period 1961–2020, following the methodology described in the Supplementary Information, section S4.3). The multivariate approach using bivariate copulas (Tootoonchi et al. 2022) better reflects the compound risk of warm temperatures (high evaporation) and low precipitation (AghaKouchak et al. 2015a).

#### Integrating soft and hard data—a combined approach

We related the perceived drought severity in each municipality to the actually observed municipality-specific hydrological severity of the 2017 and 2018 drought conditions using the non-parametric Spearman’s rank correlation coefficient *ρ*_s_ (Spearman 1904). Spearman rank correlation was chosen over linear Pearson product-moment correlation (Pearson 1920) because Spearman assumes only monotony without making prior assumptions about the nature of the relationships (e.g., linear or logarithmic).

In addition, perceived and observed hydrological severity of the droughts were visually compared to each other to identify consistent over- or underestimations in the individual perceptions.

#### Limitations

To analyze the data for a potential non-response bias, we categorized all municipalities by their observed precipitation drought category (i.e., SPI6 for 2018) and then checked the response rates across the different categories. The response rate was rather stable for most drought categories, except for the most extreme droughts. Municipalities hit by mild, moderate, and severe droughts had response rates of 37, 35, and 39%, respectively. However, out of the eleven municipalities with extreme droughts, nine (82%) answered the survey. This means municipalities heavily affected by droughts were more inclined to respond to the survey. They are only a small proportion (*N* = 9) among all survey respondents (*N* = 118). But this means that for analyzes that do not distinguish between drought severity categories, results may slightly overestimate the overall drought experience among Swedish municipalities and may therefore statistically not be entirely representative for all of Sweden’s municipalities. Nonetheless, the results provide important empirical insights on drought risk perceptions and managements among municipalities in Sweden.

## Results

### Municipalities’ understanding of droughts

Our survey revealed that out of all the municipalities that responded to the survey, 81% did not have an operational drought definition, while 15% did not know for sure. Only 4% (5 respondents) indicated to have an operational drought definition in place, either based on a threshold for groundwater or reservoir levels, or on the supply–demand relationship.

**Fig. 1:**
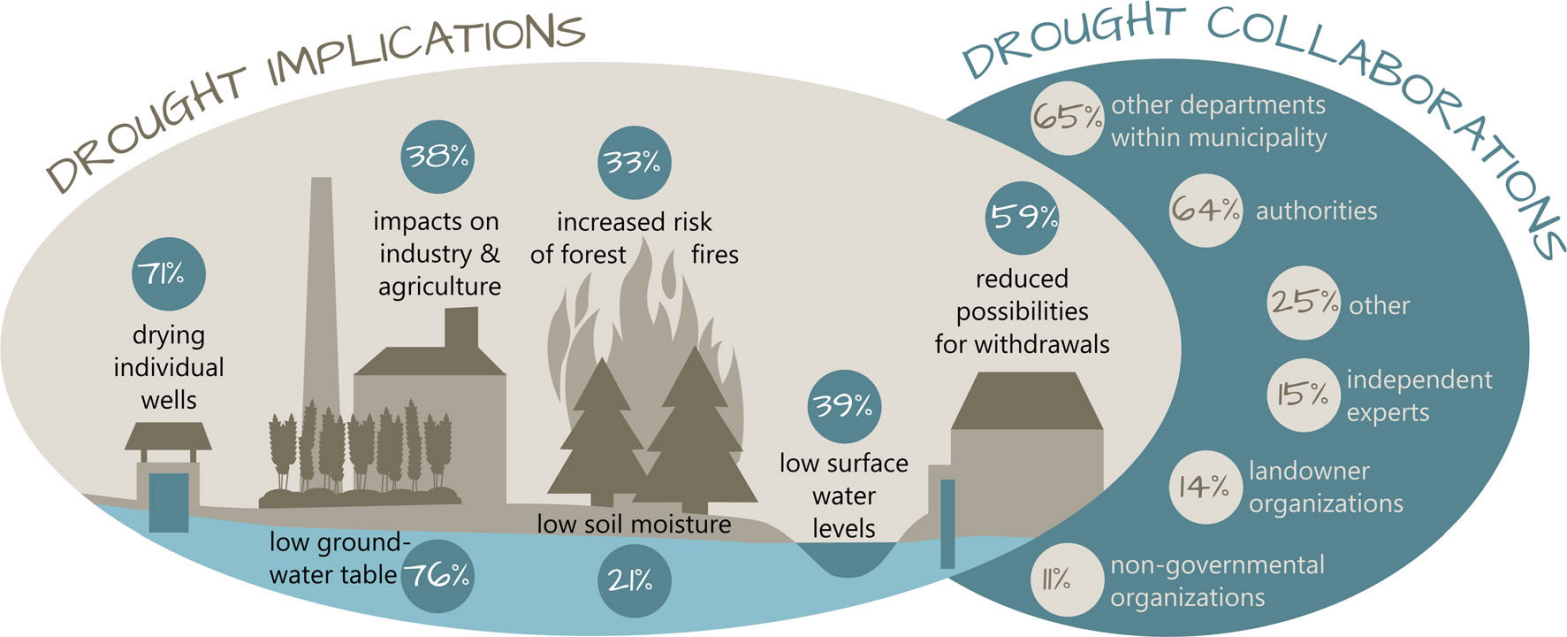
Implications of a drought situation for municipalities (left panel) and a summary of collaboration activities for drought work (right panel)

The respondents associated drought conditions with different types of implications: low groundwater levels (76%) and dry individual wells for residents (71%) were mentioned most frequently. In contrast, increased risk for forest fires (33%) and low soil moisture (21%) were mentioned least often (see Fig. 1 for detailed results).

### Municipalities’ water management

Among the surveyed municipalities, groundwater is the most frequently used water source for drinking water production in Sweden (38%), followed by surface water (29%). Several other municipalities use groundwater recharged artificially through infiltration of surface water (17%). A few municipalities use a combination of both ground- and surface water sources (7%), while only a small fraction (5%) has no own water resources and buys the water from another nearby municipality. Kindly note that these numbers correspond only to the percentage of municipalities indicating to rely on these water resources, but do not provide an indication of the withdrawn water volume. In fact, according to Statistics Sweden (2020), the largest volumetric fraction of total municipal water comes from surface water (59%), followed by 23% groundwater and 18% artificial infiltration. This discrepancy is mainly the result of a few highly-populated municipalities (i.e., Sweden’s largest three cities Stockholm, Gothenburg and Malmö) accounting for nearly half of Sweden’s municipal water withdrawal while relying on surface water.

When it comes to the municipalities’ preparedness, 35% of municipalities indicated to have a flood action plan. Even less (14%) said that they had an action plan for droughts, and the vast majority of municipalities specifically stated not having an action plan for droughts in place (72%).

The survey also revealed that the extent of collaboration in municipalities’ drought work varied substantially in terms of choice of potential collaborators (Fig. 1): while more than half of the respondents indicated to collaborate with other municipal (65%) or external authorities (64%), there seemed to be only little collaboration with independent experts (15%), landowner organizations (14%), or NGOs (11%).

### Municipalities’ planning for the future

A large fraction (81%) of the municipalities considered future climate impacts on their water resources in their every-day work, while, 12% did not consider that and 7% did not know. Most respondents agreed on that floods and drought hazards are on average increasing, and indicated either strong increase (17 and 16% of respondents, respectively for floods and droughts) or increase (68 and 81%, respectively). A larger fraction of respondents (15%) believed flood hazards would not change, while only 3% assumed drought hazards would not change.

The municipalities that assumed flood hazards would not change were spread across the entire country, except for the most extreme south and north, while municipalities that assumed drought hazards would not change were mostly located in central and northern Sweden. The vast majority of municipalities that assumed a strong increase in either flood or drought hazards were located in southern Sweden (below 60°N), which is characterized by a warmer and drier climate, as well as a higher population density. Indeed, municipalities with higher population density perceived stronger future increases for either flood or drought hazards (on average 363 and 280 inhabitants per km^2^ for floods and droughts, respectively), compared to less densely populated municipalities.

Three quarters of the respondents (75%) indicated to either have a plan/strategy or to have enough water supply to cope with a changing climate and population increase in the long run. The municipalities planned to (1) increase the capacity within the municipality (21% of respondents), e.g., through a new/larger water supply or more wells, (2) improve/renew existing infrastructure (18%), e.g., through construction of new drinking water plants or the introduction of new technology to recycle water, (3) set up an emergency water supply (18%), (4) introduce new plans and policies, including a plan for climate adaptation (15%), (5) establish new water protection areas (13%), and (6) increase collaboration especially with neighbor municipalities (14%). Only 3% of the respondents mentioned other activities such as increasing the competence within the municipality, reducing water demand, and providing more information to the public.

### Perception of the 2017 and 2018 droughts

#### Overall severity, impact, preparedness, and management

**Fig. 2:**
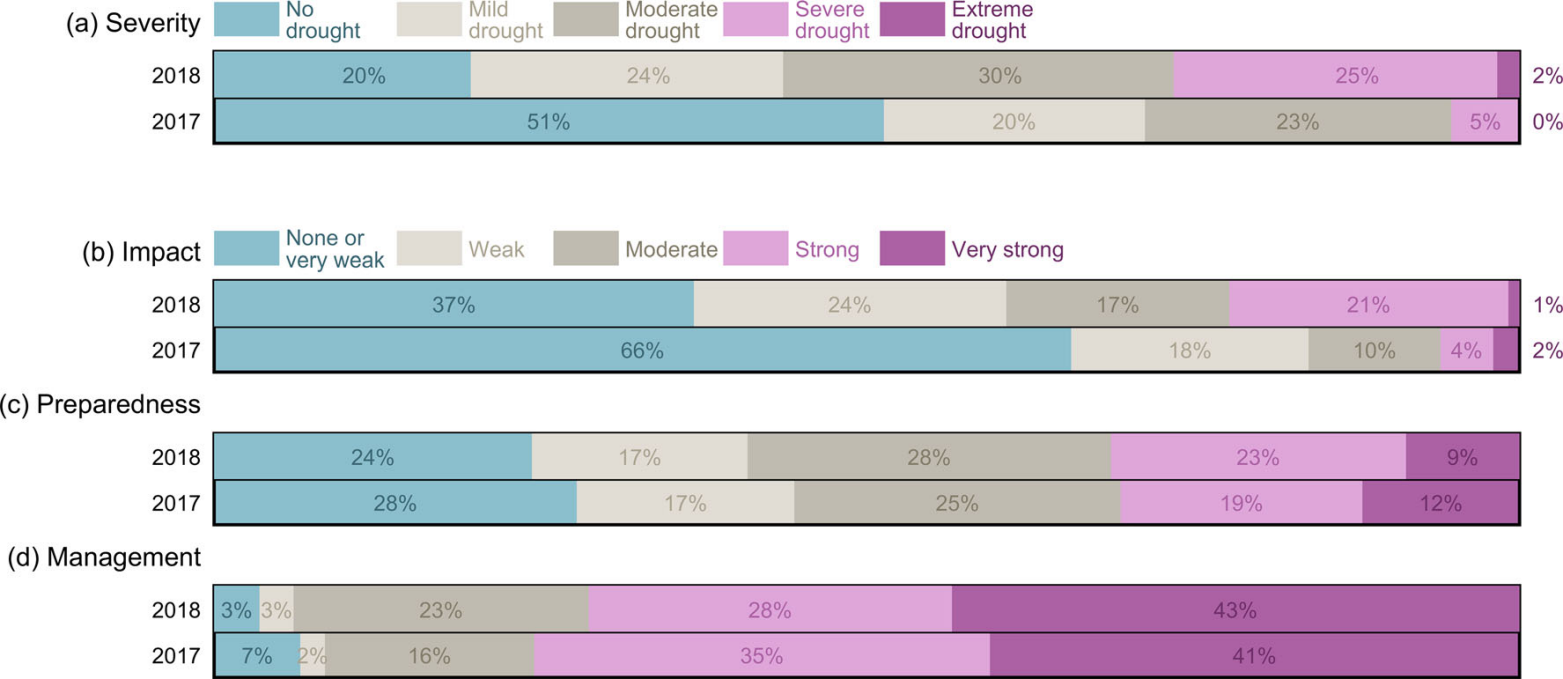
Perception of the 2018 and 2017 drought events evaluated by different measures: **a** drought severity, which ranges from no drought to extreme drought as well as **b** drought impacts, **c** preparedness, and **d** management, which are all displayed on a scale from none to very strong

The 2018 drought was perceived as much more *severe* than the 2017 drought event (Fig. 2a). With the majority of municipalities (~ 80%) classifying 2018 as a drought situation, the 2017 drought (only perceived in 49% of municipalities) was perceived on average one category less severe (significant with *p* = 1.3·10^–7^). The 2018 drought was also perceived to have stronger *impacts* compared to the 2017 drought (Fig. 2b). While 66% of municipalities reported no impacts in 2017, only 37% reported no impacts a year later. It is noteworthy that fewer municipalities experienced strong or very strong impacts in 2018 (22%) in direct comparison to the number of municipalities that perceived this year to be a severe or extreme drought (27%), while this number is rather similar for 2017 (6 versus 5%). With a Spearman correlation of *ρ*_s_ = 0.62, the perception of impacts was significantly correlated to the perceived severity of the drought events (significance level *α* = 0.05). Respondents felt on average better *prepared* in 2018 than in 2017 (Fig. 2c). For 2017, 28% reported not to have been prepared, while this number decreased somewhat for 2018 (24%). Despite the 2018 drought being perceived as much more severe and impactful than the 2017 drought, respondents indicated that they perceived the *management* in 2018 as slightly better than in 2017 (Fig. 2d). The number of municipalities who rated their own management as none or weak decreased from 9% in 2017 to 6% in 2018, while municipalities that perceived their management as very strong increased from 41 to 43%. The perceptions of preparedness and management were significantly correlated (*ρ*_s_ = 0.42) at *α* = 0.05.

The results of our analysis also demonstrate differences in the perception of different drought features and societal planning and action toward droughts. The proportion of municipalities that felt they managed both drought events well or very well was much higher (71–76%) than the proportion of municipalities that felt strongly or very strongly prepared (31–32%). The latter, in turn, was again much higher than the proportion of municipalities that experienced strong or very strong impacts (6–22%) or a severe to extreme drought (5–27%).

#### Regional differences

**Fig. 3:**
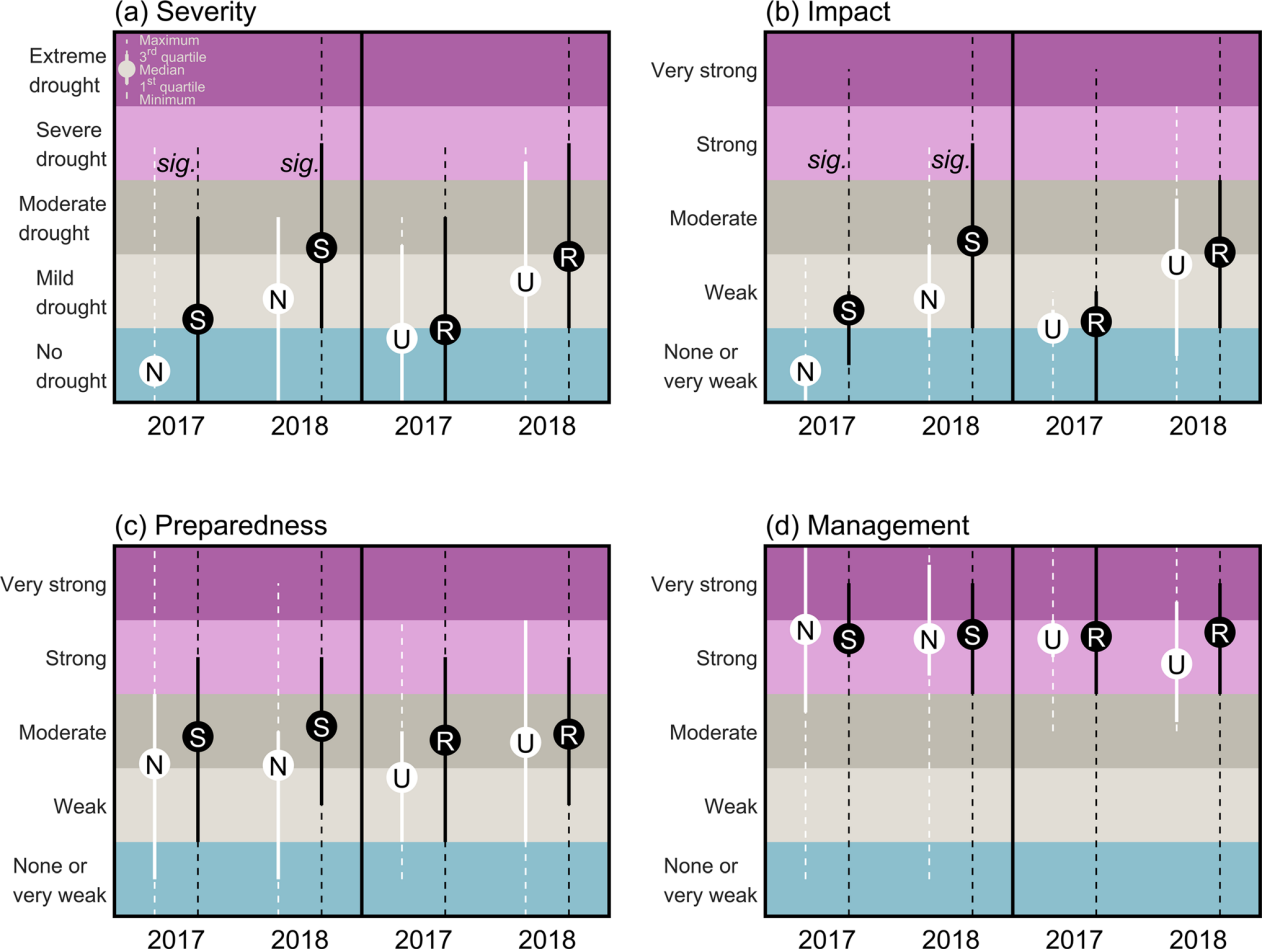
Regional differences in the perception of the 2017 and 2018 drought events evaluated by different measures: **a** drought severity, ranging from ‘no drought’ to ‘extreme drought’ as well as **b** drought impacts, **c** preparedness, and **d** management, which are all displayed on a scale from ‘none or very weak’ to ‘very strong’. Each subplot is divided into two panels, where the left panel shows a direct comparison of northern (white) versus southern (black) municipalities and where the right panel provides an overview of urban (white) versus rural (black) municipalities. Differences between regions (i.e., north/south and urban/rural) were tested for significance at a 5% level (*p* < 0.05), based on the Wilcoxon rank sum test and labeled with “sig.” accordingly

Our analysis revealed regional differences (Fig. 3), of which only the perceived *severity* (Fig. 3a, left) and the perceived *impacts* (Fig. 3b, left) between municipalities in the North and South were significant for both 2017 and 2018 at the significance level *α* = 0.05. Respondents from northern municipalities above 60°N perceived the 2017 and the 2018 drought on average as one category less severe and impactful than respondents from southern municipalities below 60°N. Also, respondents from urban areas perceived both drought events as less severe and impactful than respondents from rural areas, but these differences were not significant (Fig. 3a, b, right).

Although respondents from southern municipalities rated their own preparedness for both events in 2017 and 2018 higher than respondents from northern ones (Fig. 3c, left), and similar patterns were found between rural and urban municipalities (Fig. 3c, right), these regional differences were not significant. Similarly, no significant regional differences were found between the perception of management (Fig. 3d).

**Fig. 4:**
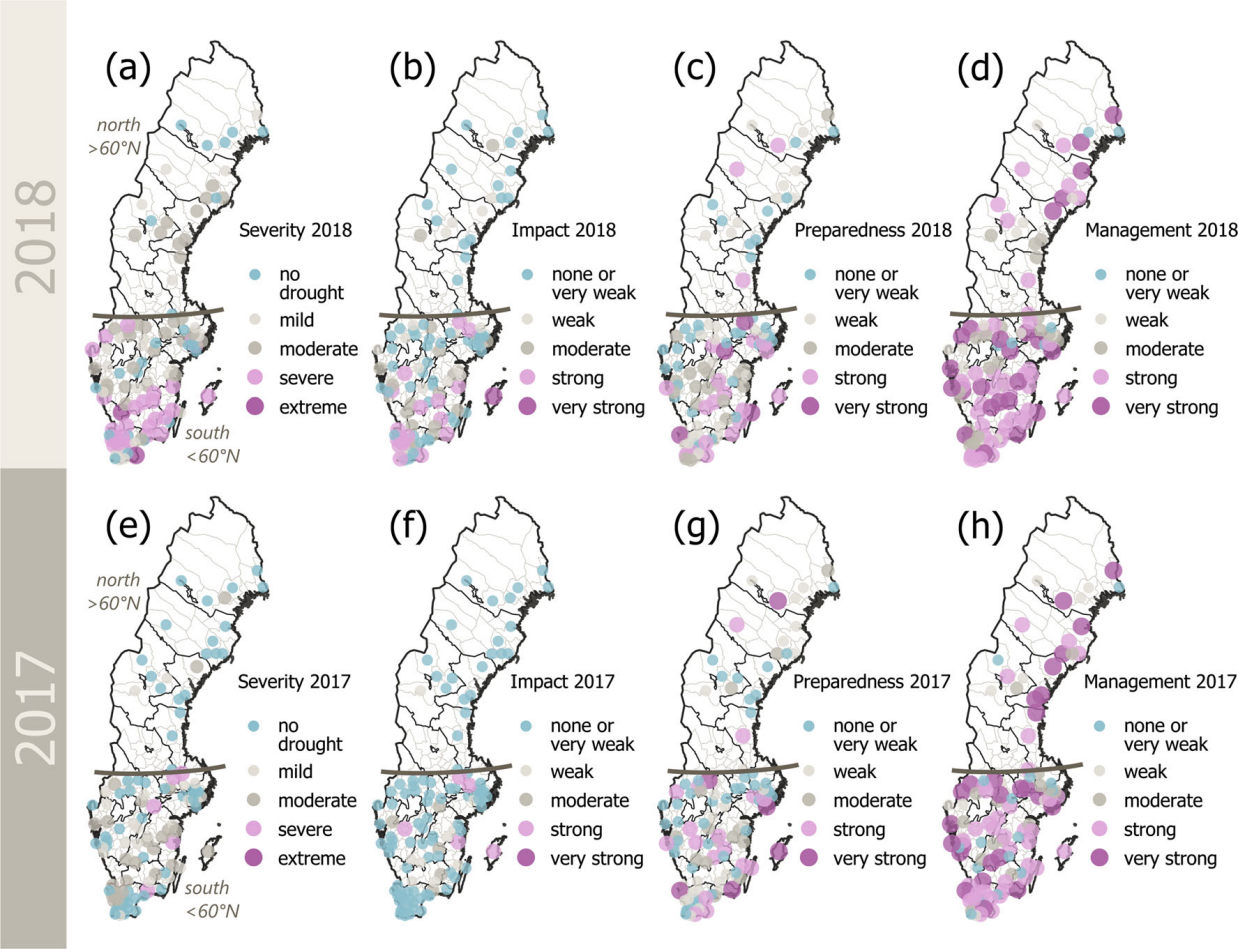
Comparison of the 2018 (upper row, a-d) and 2017 drought (lower row, e–h) perception. **a** and **e** show the perceived drought severity, ranging from no drought conditions to mild, moderate, severe, and extreme drought. **b** and **f** display the perceived impacts, **c** and **g** the perceived preparedness, and **d** and **h** the perceived management, all ranging from none or very weak to very strong

The described regional patterns were also confirmed when analyzing the spatial distribution of survey responses in more detail (Fig. 4). The 2018 drought was perceived as much more severe (Fig. 4a) than the 2017 drought (Fig. 4e), especially in southern and central parts of the country. A similar spatial pattern occurred for the perceived impacts in 2018 (Fig. 4b) and 2017 (Fig. 4f). Municipalities farthest north did not perceive drought impacts at all in both years, while respondents from municipalities in the central parts of the county perceived very weak to moderate impacts in 2018 (Fig. 4b) and no (or only very weak) impacts in 2017 (Fig. 4f). The perception among respondents from southern municipalities was much more diverse, ranging from no impacts to strong impacts, and the municipality of Gotland (an island located in the Baltic Sea in southeastern Sweden) even indicating very strong impacts in 2018 (Fig. 4b).

On the other hand, this north–south gradient was not as pronounced when it comes to the perceived level of preparedness (Fig. 4c) and management (Fig. 4d). Interestingly, municipalities in the South that experienced more severe droughts and consequent impacts generally judged both their drought preparedness level and management to be above average and, in fact, better in 2018 (Fig. 4c, d) than in 2017 (Fig. 4g, h).

#### Sectoral drought impacts and their countermeasures

According to the respondents, the drought periods had certain effects on several different sectors, with drinking water supply being mentioned most often (54% of responses in 2018 and 30% in 2017), followed by agricultural irrigation (40 and 18%, respectively) and available water for livestock (40 and 11%). Industry (3–4% both years), tourism (6 and 3%), and forest industry through forest fires (14 and 3%) were mentioned less frequently. When referring to drinking water supply, various respondents highlighted in free-text responses that the public drinking water supply was not affected, while households with private wells were considerably impacted by the drought events. In general, respondents indicated that the 2018 drought impacted a larger number of sectors (on average 1.6, with a standard deviation of 1.3) than the 2017 drought (on average 0.7 sectors, with a standard deviation of 1.0).

On average, the respondents specified that two drought measures were implemented in 2018 and only one in 2017. In 2018, the most frequently employed measure was the introduction of an information campaign targeted toward the general public (mentioned by 66% of respondents), followed by regular measurements of water levels and drought monitoring (62%), and contacts with relevant authorities (38%). These were also the most frequent actions in 2017, but implemented to a much lesser degree by approximately only half as many municipalities. Other measures mentioned were related to reducing water withdrawals (29% in 2018 and 8% in 2017), assistance offered to parts of the municipality (25 and 11%, respectively), and collaboration with other municipalities (23 and 18%).

### Link between perceived drought risk and drought management

Only 14% of respondents indicated that their municipality had a drought action plan in place at the time of the survey. All of these municipalities were located in southern Sweden. Respondents from municipalities with a drought action plan perceived a significantly stronger severity, as well as better preparedness in both 2017 and 2018, as compared to municipalities without the plan (Table 1). While the difference in the perceived impacts between both groups was not significant in 2017, in 2018 respondents perceived significantly stronger impacts in the municipalities with drought action plans (Table 1). Although this seems to contradict the idea that an action plan lowers vulnerability and subsequently impacts, the effects of action plans are likely overshadowed by drought severity, which was perceived as much stronger in the municipalities with action plans (Table 1) as they are all located in the more severely affected South. This is also reflected in the number of affected sectors, which differed significantly between municipalities with and without a drought action plan (on average 1.6 sectors in 2017 versus 2.4 in 2018 compared to 0.6 respective 1.5). Moreover, municipalities with a plan adopted 2 to 2.5 more measures than municipalities without an action plan (Table 1).

**Table 1:** Characteristics of perceived drought values separated by year of drought (2017 versus 2018) and by municipalities *with* and *without* a drought action plan. *p*-value s of the Wilcoxon rank sum test are shown as well, with bold italic values with an asterisk (*) denoting significant differences in the medians of the two groups (i.e., municipalities with and without plans) at the significance level *α* = 0.05

Perceived drought values	2017			2018		
	With action plan	Without action plan	*p*-value	With action plan	Without action plan	*p*-value
Severity	Moderate	No drought	0.041*	Severe	Mild	0.001*
Impact	None/very weak	None/very weak	0.862	Strong	Weak	0.025*
Preparedness	Moderate	Weak	0.007*	Moderate	Weak	0.023*
Management	Strong	Strong	0.123	Strong	Strong	0.903
No. of affected sectors	1.6	0.6	< 0.001*	2.4	1.5	0.007*
No. of measures	3.4	1.4	< 0.001*	4.9	2.4	< 0.001*

With the majority of the respondents indicating that they actively consider a changing climate in the coming future (81%) and that they believe drought risk will increase 97%) in a future climate (see Sect. 4.3 Municipalities’ planning for the future), one might assume that these municipalities were more likely to have a drought action plan in place. However, given the generally high number of municipalities that assume increasing drought risks and the low number (14%) of municipalities with a drought action plan (see Sect. 4.2 Municipalities’ water management), there was no statistical evidence to support this assumption.

Furthermore, respondents that perceived strong or very strong impacts (27%) indicated higher intentions to develop a drought action plan compared to those that perceived less strong impacts (18%). However, the difference between these two groups was statistically not significant (*p*-value = 0.43 at *α* = 0.05; Fisher’s exact test).

### Integrating soft and hard data—perceived versus observed drought severity

#### Hydroclimatic analysis

**Fig. 5:**
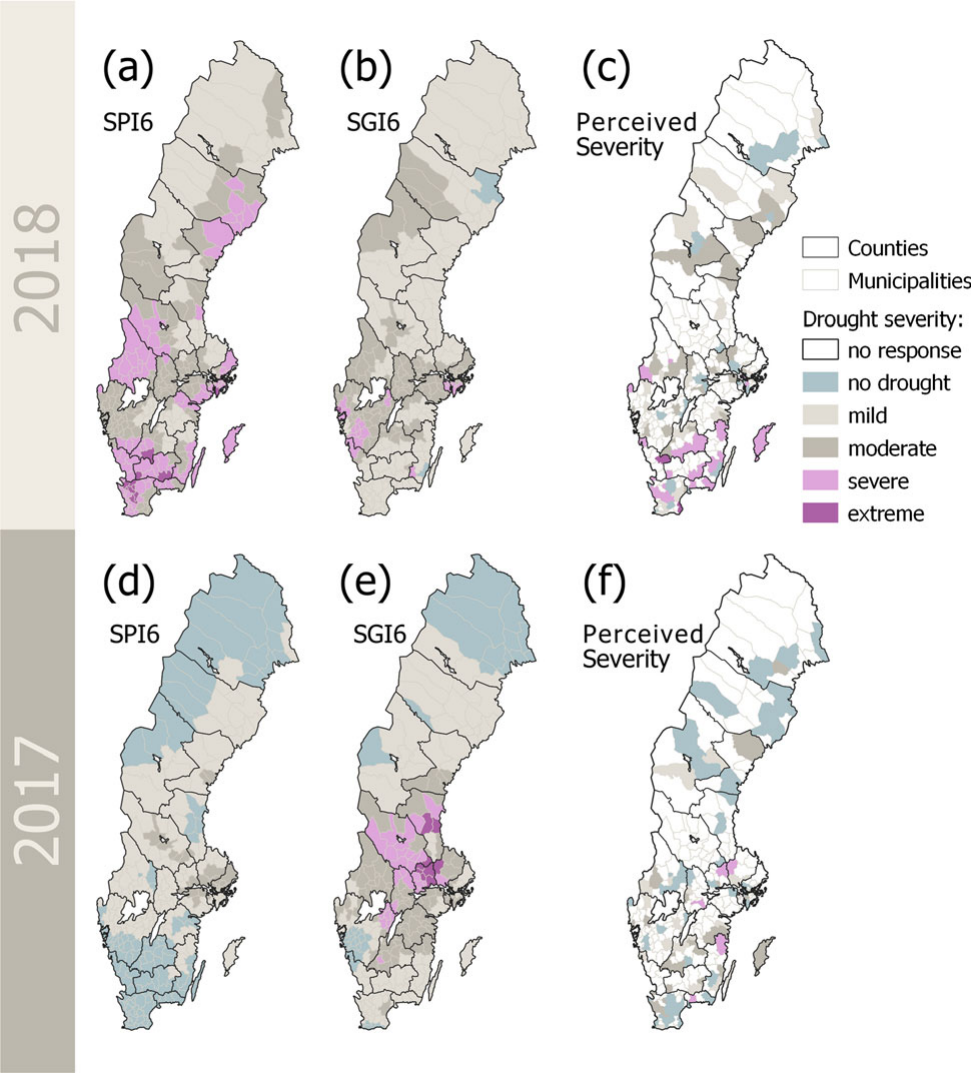
Comparison of the 2018 (upper row, **a**–**c**) and 2017 (lower row, **e**, **f**) precipitation deficits as expressed by the SPI6 in August (**a** and **d**), the groundwater deficits as expressed by SGI6 in August (**b** and **e**) and the perceived drought severity according to the survey respondents

The analysis of the standardized precipitation index (SPI) revealed that the droughts of 2017 and 2018 were wide-spread moderate to severe drought events (Fig. 5a, d). In August 2018, all municipalities were in a drought state (Fig. 5a). Only 21% of the municipalities suffered from a mild, while the rest experienced either a moderate (41%), severe (33%), or even an extreme (4%) precipitation drought. Southern municipalities suffered on average from more severe drought conditions than northern municipalities (Fig. 5a). Similar patterns could also be observed in the groundwater deficits (Fig. 5b, e): all except one municipality suffered from such deficit in 2018 (Fig. 5b). With 52% of municipalities suffering from mild, 39% from moderate, and only 8% from extreme groundwater drought, the groundwater deficit (Fig. 5b) was generally less strongly pronounced (i.e., less severe drought conditions) than the precipitation deficit.

In comparison, year 2017 was hydrologically much less severe. According to the SPI6 for August 2017, 42% of the municipalities were not in drought state, while 45% were suffering from mild and 11% of the municipalities from moderate precipitation deficits (Fig. 5d). The 2017 drought had a limited spatial extent, mainly hitting central Sweden, while the most southern and northern parts were not affected (Fig. 5d). This event was mainly caused by low winter precipitation, while the summer precipitation was on normal levels and was able to compensate for the winter deficits in many parts of the country (Fig. 5d). Thus, the return period for the March-August precipitation deficit was as low as 1.5 years. But it should be highlighted that the propagation of precipitation deficits into groundwater typically takes considerably longer time. Thus, the precipitation deficits earlier that year were reflected in the summer groundwater deficits (Fig. 5e), which were mild in 42% and moderate in 30% of the municipalities. Especially central Sweden suffered from severe (11%) and extreme (3%) groundwater droughts in 2017 (Fig. 5e).

**Fig. 6:**
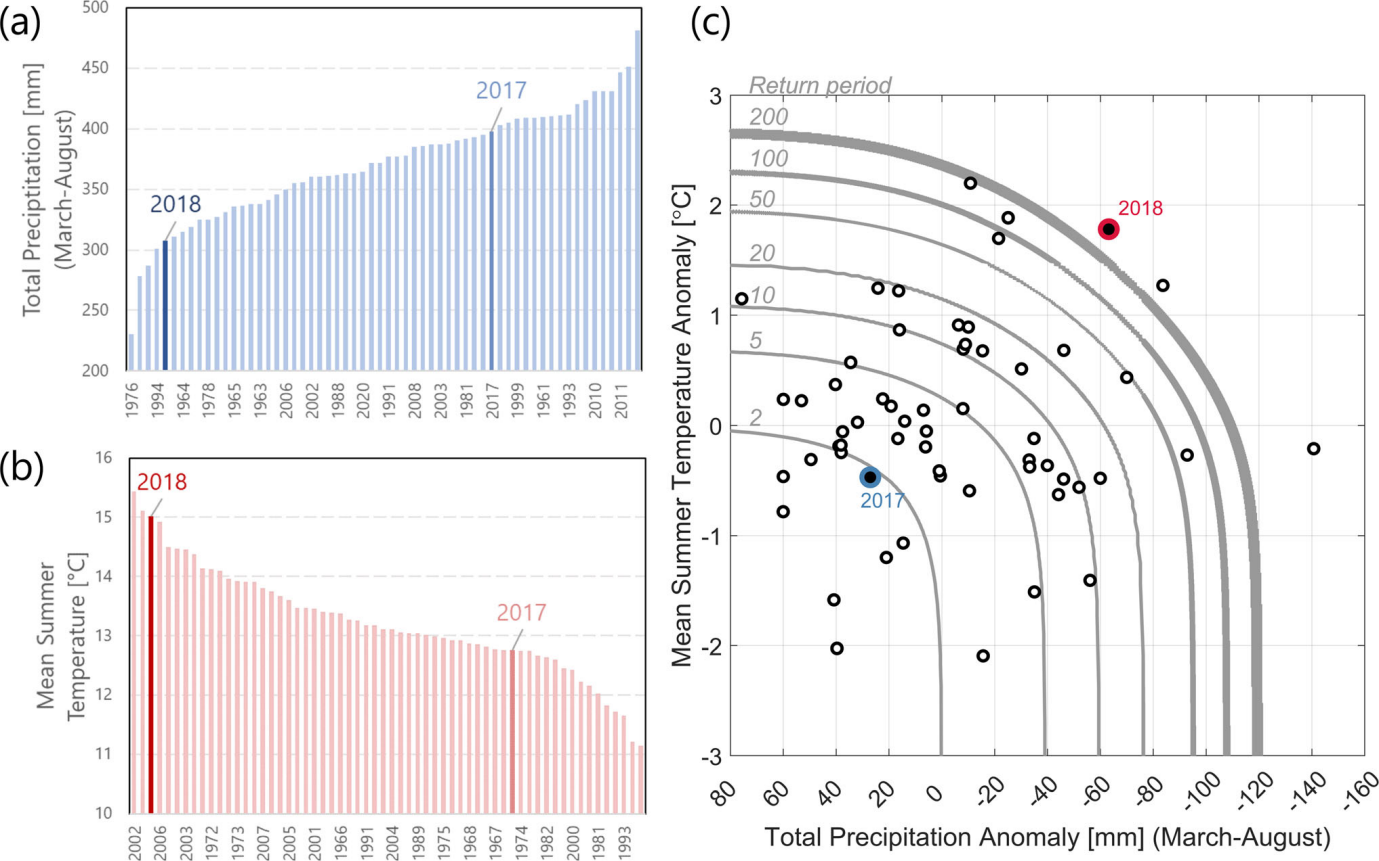
Ranked historical **a** total March–August precipitation and **b** average summer temperature averaged over entire Sweden in direct comparison to **c** their concurrent return period based on data from 1961 to 2020. In **c**, historical observations are shown as circles, while the return periods are shown as black isolines. Events in the upper-right corner correspond to a warm (*y*-axis) and dry (*x*-axis) condition. Both years (2017 and 2018) are highlighted

Due to a relative local hazard, 2017 did not stand out as a particularly dry (Fig. 6a) or hot (Fig. 6b) summer when averaged over the entire country. This was, however, different for the 2018 drought: if we consider only precipitation deficits, the return period for the 2018 summer drought was on average 12 years, ranking fifth among all years from 1961 to 2020 (Fig. 6a), while the return period was 20 years (ranking third) if we consider only temperature anomalies (Fig. 6b). Utilizing the multivariate copula approach to estimate the concurrent probability of low precipitation and high temperatures, the 2018 drought appeared to be a much more extreme and rarer event with a return period of little over 200 years (Fig. 6c). Comparable extreme conditions only occurred in 1969 (less extreme temperature, but drier conditions) and in 1976 (normal temperatures, but extremely dry conditions).

#### Comparison between observed and perceived severity

The perceived drought severity, as revealed in the survey, did not always match the observed severity of meteorological (i.e., precipitation) or groundwater droughts (Fig. 5c, f). In fact, the only consistently significant correlations (at 5% significance level) were found between perceived severity and SPI6 in 2018, while there were only few significant correlations between perceived severity and SGI6 (Table 2).

**Table 2:** Spearman Rank Correlation between perceived severity (variable 1) and observed precipitation and groundwater deficits (variable 2) during the summer drought events of 2017 and 2018. Correlations are estimated for all municipalities as well as for sub-sets of the data categorize into different regions (i.e., north versus south and urban versus rural). Significant correlations are highlighted in italic bold and marked with an asterix (*)

Variable 1	Variable 2	Event	Municipalities	Correlation (Spearman *ρ*)	*p*-value
Perceived severity	SPI6 August	2017	All	− 0.02	0.824
			North	− 0.01	0.969
			South	0.05	0.603
			Urban	− 0.30	0.375
			Rural	0.01	0.904
		**2018**	***All***	***0.38***	< 0.001*
			***North***	***0.49***	0.012*
			***South***	***0.32***	0.002*
			***Urban***	***0.72***	0.012*
			***Rural***	***0.36***	< 0.001*
	SGI6 August	2017	***All***	***0.20***	0.030*
			North	− 0.01	0.945
			South	0.20	0.062
			Urban	0.30	0.378
			***Rural***	***0.19***	0.049*
		2018	All	− 0.13	0.149
			North	− 0.03	0.900
			***South***	− ***0.26***	0.014*
			Urban	0.02	0.945
			Rural	− 0.14	0.147

**Fig. 7:**
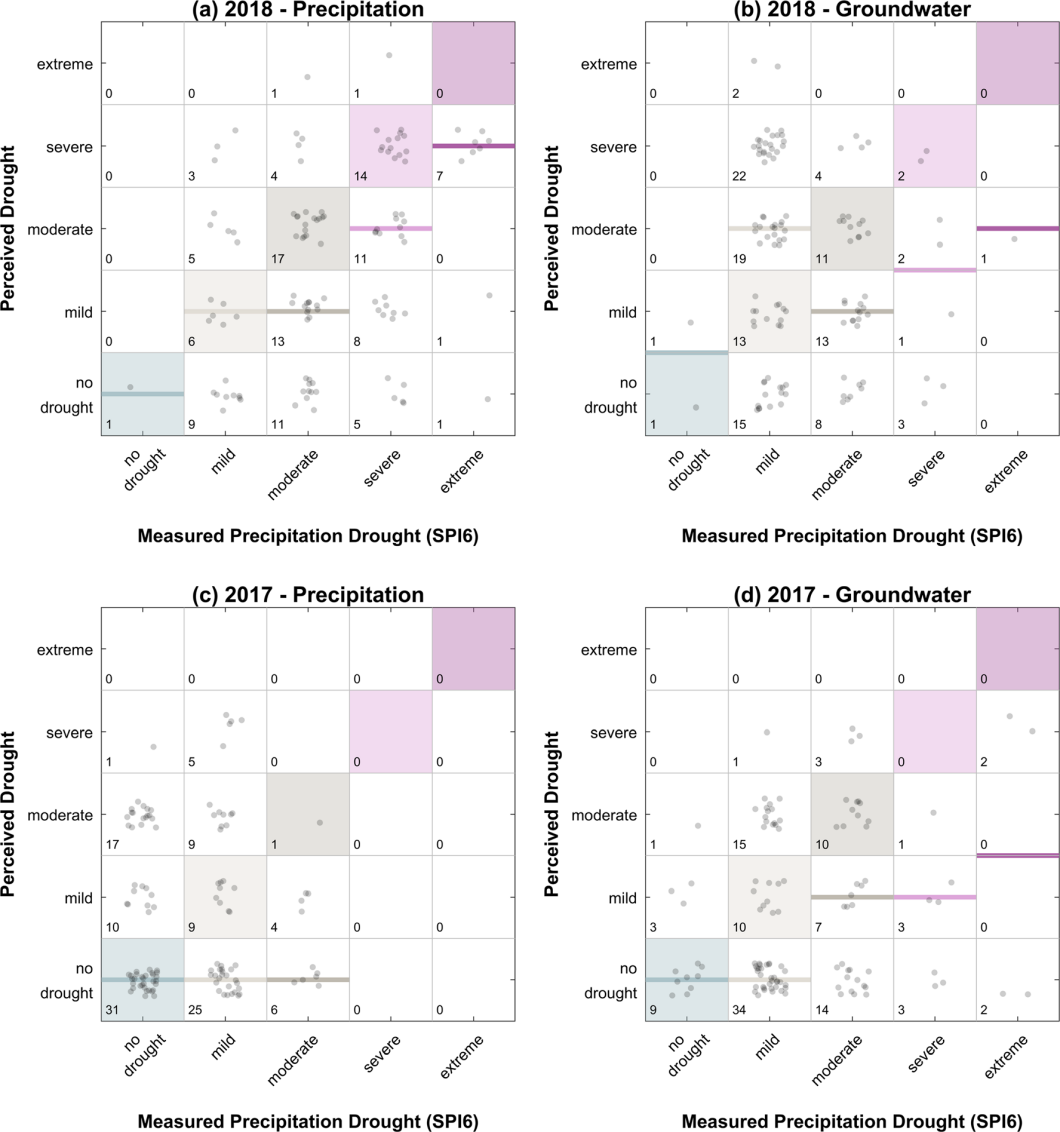
Comparison of the 2018 (**a** and **b**) and 2017 (**c** and **d**) perceived drought severity (*y*-axis ranging from normal conditions to mild, moderate, severe, and extreme) in direct comparison with actual hydrological conditions (*x*-axis), including precipitation drought severity as measured by SPI6 (**a** and **c**) and actual groundwater drought severity as measured by SGI6 (**b** and **d**) for the spring–summer period (April–September) for all municipalities (points). In a perfect world, the stakeholder perception would match the hydrological conditions and be located within the diagonally placed colored squares. Points located above those colored squares indicate that stakeholders overestimated the actual severity, while points below represent an underestimation. The colored lines show the median of all responses within one column (i.e., the median of all stakeholder perceptions for each hydrological drought category). If a median line is located above one of the colored squares, then the majority of stakeholders overestimated the hydrological drought severity, while an underestimation of the majority is shown by a line below

When directly comparing the perceived drought severity with the SPI6 and SGI6 in each municipality, a common pattern was revealed, showing that local practitioners underestimated the drought severity in municipalities that were suffering from moderate to extreme drought conditions across both years and both hydrological measures (Fig. 7a–d).

## Discussion

This is the first study in a Nordic country that systematically synthesized drought perception by relevant stakeholders based on a nationwide survey, and at the same time compared these perceptions with observed drought conditions.

Our analysis highlighted large variability in terminology and local definitions of droughts, impacts, water management, and planning for a changing future climate. The lack of general drought definitions and nationwide operational declaration schemes showcases current barriers to drought risk planning and management at the local level in Sweden. While other countries like Australia or the United States learned their lessons from previous drought events (cf., Botterill and Hayes 2012) and implemented early warning systems in combination with triggers that activate particular drought responses (Steinemann 2003), Sweden lags behind this development, even though several sectors including hydropower production, agriculture, and forest industry largely depend on a sufficient quantity of water (Lehner et al. 2005; Anderegg et al. 2013). While Tsakiris et al. (2013) highlight the importance of a clear operational definition, the key concept of droughts (i.e., a temporary severe water deficiency over a specific period and region) introduces subjectivity in defining thresholds for severity and deficiency, as well as in setting the scales for time and space (Rossi et al. 2003). Therefore, Quiring (2009) recommends the development of objective methods for establishing operational drought definitions. However, creating a universally formalized definition of drought that applies to all municipalities and sectors in Sweden may be challenging (Lloyd-Hughes 2014), especially because different policies and authorities govern different sectors (Blauhut et al. 2021). Instead, Lloyd-Hughes (2014) argues for local drought definitions that holistically consider water supply, demand, and management.

Ek et al. (2016) pointed out that several factors such as human resources, available knowledge, and financial constraints at the municipal level largely affect the risk management at local levels. Consequently, awareness of future drought risks might also be an important factor further limiting current developments in the arena of drought risk management. However, while most survey respondents indicated that they believe drought risk will increase in the future, very few municipalities had operational drought action plans, and many respondents did not see a need to prioritize development of such plans. This is problematic as the establishment of drought action and management plans prior to drought events has been identified as a powerful tool to lower vulnerability to droughts (Wilhite et al. 2000; Vogt et al. 2018; UNDRR 2021). It also implies that they will continue to rely on an emergency response in case of droughts, as during the droughts of 2017 and 2018. Although short-term measures can be effective to reduce immediate drought impacts, introducing long-term measures is essential in the face of climate and demographic changes (AghaKouchak et al. 2015b). Respondents listed a variety of suggestions for such long-term measures, which can either be categorized as (1) increasing supply or as (2) managing/reducing demand (Inman and Jeffrey 2006; Alias et al. 2017). Most of the measures mentioned belonged to the first category, focusing either on more or new water resources, new investments for new drinking water plants with higher storage and pumping capacity, or on establishing an emergency water supply. In contrast, measures to reduce water demand (e.g., information campaigns for the public, water reduction measures and reducing water losses) were mentioned much less often, which is somewhat contradictory to recent trends in sustainable water resources management. In fact, Gleick (2000) argued already two decades ago that the world has been experiencing a ‘changing water paradigm’, referring to shifts in the dynamic process of water resources management from focusing solely on tapping new water resources for supply toward addressing the management of new demands, emphasizing the “ethics of sustainability.” Sustainable water use is ‘the use of water that supports the ability of human society to endure and flourish into the indefinite future without undermining the integrity of the hydrological cycle or the ecological systems that depend on it’ (Gleick 2000). Thus, rather than exploiting new water resources to meet future needs, it is essential to understand how current and future human water demand can be met with the water presently available, while guaranteeing the preservation of ecosystems and biodiversity that are vital for human well-being (Blicharska et al. 2019; Pecl et al. 2017). Consequently, there is growing consensus that an Integrated Water Resource Management (IWRM) approach combining both the demand-side management with traditional supply activities is a more sustainable solution to cope with water shortages (Da-ping et al. 2011; Inman and Jeffrey 2006).

Overall, integrated drought risk management, which considers the entire cycle of disaster management from prediction and prevention to practical measures reducing impacts of droughts and supporting recovery in a sustainability context, is favorable over traditional emergency responses, as it creates opportunities for incorporating economic, social, and environmental pillars of sustainable development (Grobicki et al. 2015). This is not only true for developing countries with economies relying on rain-fed agriculture and pastoralism (Brüntrup and Tsegai 2017), but also valid for developed countries like Sweden (AghaKouchak et al. 2015a). Such a holistic approach also involves a meaningful participation of relevant stakeholders from different drought-prone sectors (e.g., farmer or forester's associations) and can contribute to preparedness strategies, and well-designed communication with the general public to increase their awareness and strengthen the overall capacity of the society to deal with droughts (Matti et al. 2017). However, realizing such ambitious plans and overcoming current limitations calls for substantial efforts by authorities to be able to deal with the complex nature of drought impacts and to increase Sweden’s adaptive capacity and resilience by implementing drought risk management strategies, including measures to limit hazard, exposure, and vulnerability (Buurman et al. 2017). Strategic efforts could aim to mimic the endeavors made over the past decades in relation to flood risk management. In particular, flood prevention, defense, and mitigation as well as warning, evacuation, and recovery have been on the agenda (Pettersson et al. 2017) of Swedish authorities, especially after adopting the EU flood risk directive (European Parliament and Council of the European Union 2007, p. 60). This is evident both from the integration of flood risk into Swedish law (Swedish Government 2009), but also from the survey responses that indicate that the proportion of municipalities with operational flood action plans is 2.5 times higher than the proportion with drought action plans. Similar to drought risk, the Swedish legal framework concentrates responsibility for mitigating flood risk also to the municipal level and according to Becker (2021) all Swedish municipal administrations commonly employ part- or full-time civil servants specifically working on flood risk mitigation. Additionally, the Swedish Civil Contingencies Agency (MSB) has been mapping and regularly updating the flood risk for streams and lakes since the late 1990s. Similar efforts for developing drought risk management strategies were only recently kicked off, triggered by the severe 2018 drought event, and include, e.g., the monitoring of groundwater levels and mapping of water bodies across the country.

The survey revealed high variability of perceptions of past drought events and future drought risks in Sweden, including their impacts, preparedness, and management, reflecting the country’s strong climate gradient, as well as the socio-economic heterogeneity across the country.

Impacts were perceived stronger in southern and in rural municipalities. While the southern parts generally suffered from more severe drought conditions (Teutschbein et al. 2022a), both southern and rural areas commonly depend more strongly on enough water availability than northern and urban areas, because of agriculture and the presence of private wells. A clear majority of Sweden’s agricultural areas, which is strongly water-dependent for cultivation of plants and livestock, is located in the South below 60°N (Statistics Sweden 2019), and was, thus, considerably impacted by the more severe drought conditions there (Swedish Board of Agriculture 2019). Also, rural communities in Sweden typically rely on forestry, agriculture, and mining activities, which are strongly water-dependent (Hedlund 2016). Moreover, private wells are a common way of providing individual households with drinking water (400 000 such wells in Sweden), especially in rural areas further away from the public drinking water network (Gunnarsdottir et al. 2020; Löwenhielm 2008). Additionally, the largest fraction of private wells can be found in southern regions (Maxe 2021). Thus, these households that are not connected to the public drinking supply seem particularly vulnerable to variations in the local water supply and, thus, to droughts. Similar findings have also emanated from the results of a Finnish study modeling potential effects of severe droughts on Finland’s water resources (Veijalainen et al. 2019), which reported an increased vulnerability of shallow aquifers and private shallow wells. This is a worrying result as recent reports estimate that 1.1 million people (11%) receive their drinking water permanently from these small and unregulated private wells (Gunnarsdottir et al. 2017).

Despite most municipalities not having drought action plans implemented and many households having suffered from the 2018 drought event, our results indicated a surprising confidence in relation to local preparedness levels and even more so in the management of droughts, beyond what would have been expected from the perceived drought severity and impacts.

In an attempt to explain this dichotomy, we hypothesize that municipalities that perceived severe drought conditions perhaps had to put in more efforts (i.e., more complex and time-consuming management tasks) to handle the situation and, thus, perceived a higher (better) management level. This hypothesis is supported by the fact that respondents that indicated a good management in their municipality did not necessarily perceive a high level of preparedness. Another potential explanation for this dichotomy might be a cognitive bias in the perception of the respondents (Arnott 2006), in particular a self-enhancement bias (Gosling et al. 1998), which causes individuals to rate their performance higher than a normative criterion would suggest. This type of cognitive bias is not uncommon for disasters that necessitate emergency management arrangements (Comes 2016), but is problematic and continues to challenge the application of good decision-making principles (Brooks et al. 2020).

Our analysis also revealed that there seems to be a learning effect, resulting in an increased preparedness for and improved management of the 2018 drought compared to 2017. It is common for natural disasters to trigger an assessment of the existing governance system (Lumbroso and Vinet 2012; November et al. 2007; Raikes et al. 2019) and to lead to a process of governance learning (Brody et al. 2009) that results in corrective measures that can foster a transition from crisis management toward risk reduction (Raikes et al. 2019). In this transition, drought action plans will likely play a key role. We found that municipalities with an existing action plan implemented larger numbers of drought response measures, which highlights the importance of developing drought risk management strategies. However, surprisingly, neither the perception of future drought risk nor the experience of recent drought events was able to explain whether municipalities already implemented or planned to implement a drought action plan. A somewhat concerning implication of these findings may be that municipalities, even when substantially affected by drought events, may not become more likely to invest in planning for future drought events. This result encourages future research to further explore the intrinsic and extrinsic motivations for municipalities to implement a drought action plan. As our study revealed a clear relationship between risk perception and population density, such efforts must also be placed in the context of cognitive/behavioral, socio-economic, and geographical factors within municipalities (Lechowska 2018; O’Neill et al. 2016) as well as traditional risk perceptions in a usually water-abundant country (Ahopelto et al. 2019), all of which might influence local perception and should be considered in awareness-raising activities.

The novelty of this paper lies in a combined approach that integrated the benefits of using both soft and hard data by augmenting the survey respondents’ drought perceptions with hydrological drought indices. The hard data allowed us to quantitatively describe the severity and dynamics of recent drought events, while a fusion with soft data was key to explain the impacts of these events and understand practitioners’ drought responses. Thus, the combined approach provided more sophisticated insights about the complexity of social and hydrological systems impacted by droughts and the cognitive abilities of mankind to understand and link them, thus generating unique empirical knowledge and opportunities for new developments and improvements in the arena of drought management and risk reduction.

Our study revealed that the 2018 drought was an outstanding event across entire Sweden that ranked among the top three of the most severe drought events over the past 60 years. Record-breaking summer temperatures across northern Europe and a lack of precipitation caused meteorological drought conditions in the Nordic countries (Bakke et al. 2020), which ultimately propagated within the hydrological system and led to serious declines in streamflow (Teutschbein et al. 2022a) and groundwater levels. While 2018 experienced a classical rainfall-deficit drought that was further exacerbated by a heatwave with high evaporation rates during the summer months (Van Loon and Van Lanen 2012), the 2017 drought was rather a long-term consequence of low ground- and surface water levels that started to emerge already in 2016, and which did not recover in the winter of 2016/2017 due to unusually warm winter temperatures and less than normal winter precipitation (Stensen et al. 2019). Such differences in drought typologies, spatial extent, and severity levels across different drought events are common as drought emergence strongly depends on large-scale weather patterns and regional differences in land-surface properties (Blauhut et al. 2021; Kingston et al. 2015).

Our results indicated that local practitioners perceived the actual severity of the droughts differently from the reality and their perceptions did not match the observed spatial patterns, which might have been caused by a multitude of factors. Smakhtin and Schipper (2008) argue that drought perception intrinsically depends on the debate surrounding their conceptualization and related terminology. Thus, we can only speculate that perhaps one factor relates to confusion in the semantics, causing the respondents to weigh in other drought aspects such as demand (i.e., rather relating to man-made water shortages instead of focusing on the drought hazard), impacts or preparedness to provide a final severity estimate. Other potential factors relate to timing and might be cognitively and neurobiologically motivated (Lifanov et al. 2021): the survey was conducted at the end of 2018, when the 2018 summer drought was still fresh in mind, while memories from the 2017 drought might have already started to fade. Thus, estimates of the most recent drought might have been more accurate than the drought event longer ago. Indeed, the fact that there are significant correlations between the perceived and observed drought severity mostly for the latest 2018 drought event and almost no correlations for the previous 2017 event indicates a quickly declining ability of practitioners to keep awareness of drought hazards high, i.e., the “collective memory” of Swedish society is short-lived (Pfister 2011; Viglione et al. 2014).

However, the mismatch between perception and observations for 2017 (SPI and SGI) and 2018 (only SGI) might also be motivated by cognitive biases such as the recency bias (Comes 2016) that causes us to favor recent events over events in the past, or the availability biases (Tversky and Kahneman 1973), which refers to judging a situation based on the ease with which it comes to mind. Consequently, practitioners seem to find it easier to relate drought severity to easily observable (and recallable) short-term processes that are visible “here and now”, e.g., like in 2018, the warm temperature or little precipitation over a limited period in summer, than relating drought severity to long-term processes that involve the preceding status of aquifers, precipitation deficits over longer time-periods and lack of snow in winter (which was the case in 2017). Thus, a potential drought monitoring and early warning system should not only rely on easy-to-measure indicators (e.g., precipitation or soil-moisture deficits) that represent shorter-term processes, but should also include indicators that represent processes in the water cycle at various spatial and temporal scales that could in the long-run also trigger droughts (Bachmair et al. 2016).

The existing disconnect between perceived and observed drought severity may lead to a distorted perception of drought risks, which could potentially hamper drought preparedness and, thus, prevent practitioners to efficiently manage droughts. Therefore, well-designed communication and education efforts are needed to increase practitioners’ and public awareness and the “social memory”. However, coordinated national efforts, including for instance drought monitoring, vulnerability, and impact assessments, early warning systems, national drought policies, and the adoption of regional drought mitigation strategies and action plans are also required to eventually cope more efficiently with drought hazards and extended water shortages (Wilhite 2019). First steps forward have been made by national agencies through the implementation of drought risk mapping tools such as SMHI’s “Risk for Water shortages” tool (SMHI 2022) or SGU’s “Groundwater levels” tool (SGU 2022), which enable a more tailored mapping of the dominating water resources (surface or groundwater) in a particular county. Nonetheless, we need to recognize the importance of overcoming main barriers relating to time, and human and financial constrains (Aguiar et al. 2018) to boost the development of a holistic approach in an attempt to strengthen the overall capacity of the society to deal with droughts (Matti et al. 2017). Based on the findings in our study, we argue that such an approach should (1) incentivize municipalities to create holistic climate-change adaptation plans, (2) consider the supply–demand balance and high losses in the drinking water supply chain, (3) integrate indicators such as SPI or SGI (i.e., hard data) into municipal planning as support for identification/classification of drought events and as potential triggers in drought early warning systems, (4) provide information and education (soft data) to practitioners to improve their perception, and (5) involve relevant stakeholders from different drought-prone sectors to co-create local/regional strategies.

## Conclusion

This study embarked to understand practitioners’ perception of drought severity, impacts, preparedness, and management at municipal level in Sweden, and how well their perception matches observed drought hazards. A mapping of the perceptions of Swedish municipalities on their local water resources, future risks of droughts, as well as their perception of recent drought events revealed a far-reaching lack of drought definitions and operational drought action plans at municipal level across Sweden. In particular, rural areas in southern Sweden with a high proportion of agricultural activities and stronger dependence on private wells for drinking water supply perceived the strongest impacts during recent drought events and would benefit most from integrating drought action plans and management strategies to lower present-day drought vulnerability. Moreover, a comparison of observed precipitation and groundwater deficits with perceived drought severity resulted in a mismatch, except for the most recent drought event of 2018 for which correlations were found. Potential contributors to these discrepancies might be a short-lived social memory, cognitive biases and a lack of harmonized drought conceptualization and terminology among practitioners. Thus, further efforts like those for improving flood risk management are urgently needed to increase practitioners’ awareness, develop a common conceptual understanding and align their perceptions of drought hazards, eventually contributing to improved risk management strategies to deal with drought vulnerability and to adapt to a changing climate also in developed countries like Sweden.

## Supplementary Information

Below is the link to the electronic supplementary material.

Supplementary file1 (PDF 733 KB)

## References

[CR2] AghaKouchak A, Cheng L, Mazdiyasni O, Farahmand A (2015). Global warming and changes in risk of concurrent climate extremes: Insights from the 2014 California drought. Geophysical Research Letters.

[CR3] AghaKouchak A, Feldman D, Hoerling M, Huxman T, Lund J (2015). Water and climate: Recognize anthropogenic drought. Nature.

[CR4] Agrawal N, Elliott M, Simonovic SP (2020). Risk and resilience: A case of perception versus reality in flood management. Water.

[CR5] Aguiar FC, Bentz J, Silva JMN, Fonseca AL, Swart R, Santos FD, Penha-Lopes G (2018). Adaptation to climate change at local level in Europe: An overview. Environmental Science & Policy.

[CR6] Ahopelto L, Veijalainen N, Guillaume JHA, Keskinen M, Marttunen M, Varis O (2019). Can there be water scarcity with abundance of water? Analyzing water stress during a severe drought in Finland. Sustainability.

[CR7] Alias AH, Boyle CA, Hassim S (2017). Water demand management: A review on the mechanisms to reduce water demand and consumption. International Journal of Civil Engineering and Technology (IJCIET).

[CR8] Anderegg WRL, Kane JM, Anderegg LDL (2013). Consequences of widespread tree mortality triggered by drought and temperature stress. Nature Climate Change.

[CR9] Arnott D (2006). Cognitive biases and decision support systems development: A design science approach. Information Systems Journal.

[CR10] Asadzadeh M, Tolson BA, Burn DH (2014). A new selection metric for multiobjective hydrologic model calibration. Water Resources Research.

[CR11] Bachmair S, Stahl K, Collins K, Hannaford J, Acreman M, Svoboda M, Knutson C, Smith KH (2016). Drought indicators revisited: The need for a wider consideration of environment and society. Wires Water.

[CR12] Bakke SJ, Ionita M, Tallaksen LM (2020). The 2018 northern European hydrological drought and its drivers in a historical perspective. Hydrology and Earth System Sciences.

[CR13] Becker P (2021). Fragmentation, commodification and responsibilisation in the governing of flood risk mitigation in Sweden. Environment and Planning c: Politics and Space.

[CR14] Blauhut V, Stoelzle M, Ahopelto L, Brunner MI, Teutschbein C, Wendt DE, Akstinas V, Bakke SJ (2021). Lessons from the 2018–2019 European droughts: A collective need for unifying drought risk management. Natural Hazards and Earth System Sciences Discussions.

[CR15] Blicharska M, Smithers RJ, Mikusinski G, Ronnback P, Harrison PA, Nilsson M, Sutherland WJ (2019). Biodiversity’s contributions to sustainable development. Nature Sustainability.

[CR16] Bloomfield JP, Marchant BP (2013). Analysis of groundwater drought building on the standardised precipitation index approach. Hydrology and Earth System Sciences.

[CR17] Botterill LC, Hayes MJ (2012). Drought triggers and declarations: Science and policy considerations for drought risk management. Natural Hazards.

[CR18] Bradford RB, Vogt JV, Somma F (2000). Drought events in Europe. Drought and drought mitigation in Europe.

[CR19] Brody SD, Zahran S, Highfield WE, Bernhardt SP, Vedlitz A (2009). Policy learning for flood mitigation: A longitudinal assessment of the community rating system in Florida. Risk Analysis.

[CR20] Brooks B, Curnin S, Owen C, Bearman C (2020). Managing cognitive biases during disaster response: The development of an aide memoire. Cognition, Technology & Work.

[CR21] Brüntrup, M., and D. Tsegai. 2017. Drought adaptation and resilience in developing countries (Research Report No. 23/2017a). Briefing Paper.

[CR22] Buurman J, Mens MJP, Dahm RJ (2017). Strategies for urban drought risk management: A comparison of 10 large cities. International Journal of Water Resources Development.

[CR23] Carlsson-Kanyama A (2013). Barriers in municipal climate change adaptation: Results from case studies using backcasting. Futures.

[CR24] Casajus Valles, A., A. De Jager, F. Dottori, L. Galbusera, B. García Puerta, G. Giannopoulos, S. Girgin, M.A. Hernandez Ceballos, et al. 2019. Recommendations for national risk assessment for disaster risk management in EU: approaches for identifying, analysing and evaluating risks: version 0.

[CR25] Comes, T. 2016. Cognitive biases in humanitarian sensemaking and decision-making lessons from field research. In *2016 IEEE international multi-disciplinary conference on cognitive methods in situation awareness and decision support (CogSIMA). Presented at the 2016 IEEE international multi-disciplinary conference on cognitive methods in situation awareness and decision support (CogSIMA)*, 56–62. 10.1109/COGSIMA.2016.7497786.

[CR26] Dakurah G (2021). How do farmers’ perceptions of climate variability and change match or and mismatch climatic data? Evidence from North-west Ghana. GeoJournal.

[CR27] Dannevig H, Rauken T, Hovelsrud G (2012). Implementing adaptation to climate change at the local level. Local Environment.

[CR28] Da-ping X, Hong-yu G, Dan H (2011). Discussion on the demand management of water resources. Procedia Environmental Sciences.

[CR29] Ek, K., S. Goytia, M. Pettersson, and E. Spegel. 2016. Analysing and evaluating flood risk governance in Sweden—adaptation to climate change? STAR—FLOOD Consortium, Utrecht, The Netherlands.

[CR30] Eklund, A., J.A. Mårtensson, S. Bergström, E. Björck, J. Dahné, L. Lindström, D. Nordborg, J. Olsson, et al. 2015. Sveriges framtida klimat - underlag till dricksvattenutredningen [English: Sweden’s climate—a basis for investigating drinking water] (No. 14), Klimatologi (Climatology). Swedish Meteorological and Hydrological Institute (SMHI), Norrköping, Sweden.

[CR31] Elfström, C. 2015. Översvämningar kostade rekordmycket [English: Floods cost a record amount]. https://www.svt.se/nyheter/inrikes/oversvamningar-kostade-rekordmycket. Accessed 28 Mar 2023.

[CR32] European Commission. 2013. Decision No 1313/2013/EU of the European Parliament and of the Council of 17 December 2013 on a Union Civil Protection Mechanism (No. 1313/2013/EU). Brussels.

[CR33] European Parliament and Council of the European Union. 2007. Directive 2007/60/EC of the European Parliament and of the Council of 23 October 2007 on the assessment and management of flood risk.

[CR1] FAO. 2020. Total renewable water resources (10^9^ m^3^/year). AQUASTAT database Database. https://tableau.apps.fao.org/views/ReviewDashboard-v1/country_dashboard?%3Aembed=y&%3AisGuestRedirectFromVizportal=y. Accessed 28 Mar 2023.

[CR34] Fink AH, Brücher T, Krüger A, Leckebusch GC, Pinto JG, Ulbrich U (2004). The 2003 European summer heatwaves and drought–synoptic diagnosis and impacts. Weather.

[CR35] Fisher RA (1922). On the interpretation of χ^2^ from contingency tables, and the calculation of P. Journal of the Royal Statistical Society.

[CR36] Fuchs S, Karagiorgos K, Kitikidou K, Maris F, Paparrizos S, Thaler T (2017). Flood risk perception and adaptation capacity: A contribution to the socio-hydrology debate. Hydrology and Earth System Sciences.

[CR37] Geological Survey of Sweden. 2017. Vattenbrist hotar stora delar av landet [English: Water shortage threatens large parts of the country]. https://www.sgu.se/om-sgu/nyheter/2017/maj/vattenbrist-hotar-stora-delar-av-landet/. Accessed 4 Aug 2018.

[CR38] Gleick PH (2000). A look at twenty-first century water resources development. Water International.

[CR39] Gosling SD, John OP, Craik KH, Robins RW (1998). Do people know how they behave? Self-reported act frequencies compared with on-line codings by observers. Journal of Personality and Social Psychology.

[CR40] Grobicki A, MacLeod F, Pischke F (2015). Integrated policies and practices for flood and drought risk management. Water Policy.

[CR41] Gunnarsdottir MJ, Persson KM, Andradottir HO, Gardarsson SM (2017). Status of small water supplies in the Nordic countries: Characteristics, water quality and challenges. International Journal of Hygiene and Environmental Health.

[CR42] Gunnarsdottir MJ, Gardarsson SM, Schultz AC, Albrechtsen H-J, Hansen LT, Gerlach Bergkvist KS, Rossi PM, Klöve B (2020). Status of risk-based approach and national framework for safe drinking water in small water supplies of the Nordic water sector. International Journal of Hygiene and Environmental Health.

[CR62] HaV. 2018. Vattenbrist och torka – så påverkar det vattenmiljön [English: Water shortages and droughts - how they affect the aquatic environment]. Havs- och vattenmyndigheten. https://www.havochvatten.se/miljopaverkan-och-atgarder/miljopaverkan/vattenbrist/vattenbrist-och-torka---sa-paverkar-det-vattenmiljon.html. Accessed 23 Mar 2023.

[CR43] Hedlund M (2016). Mapping the socioeconomic landscape of rural Sweden: Towards a typology of rural areas. Regional Studies.

[CR44] Hosking JRM, Wallis JR (1993). Some statistics useful in regional frequency analysis. Water Resources Research.

[CR45] Inman D, Jeffrey P (2006). A review of residential water conservation tool performance and influences on implementation effectiveness. Urban Water Journal.

[CR46] IPCC. 2014a. *Climate change 2013: The physical science basis, Contribution of Working Group I to the fifth assessment report of the intergovernmental panel on climate change*. Cambridge: Cambridge University Press.

[CR47] IPCC (2014). Climate change 2014: Impacts, adaptation, and vulnerability: Working Group II contribution to the fifth assessment report of the intergovernmental panel on climate change.

[CR48] IPCC (2021). Climate change 2021: The physical science basis. Contribution of Working Group I to the sixth assessment report of the intergovernmental panel on climate change.

[CR49] Kingston DG, Stagge JH, Tallaksen LM, Hannah DM (2015). European-scale drought: Understanding connections between atmospheric circulation and meteorological drought indices. Journal of Climate.

[CR51] Lechowska E (2018). What determines flood risk perception? A review of factors of flood risk perception and relations between its basic elements. Natural Hazards.

[CR52] Lehner B, Czisch G, Vassolo S (2005). The impact of global change on the hydropower potential of Europe: A model-based analysis. Energy Policy.

[CR53] Lifanov J, Linde-Domingo J, Wimber M (2021). Feature-specific reaction times reveal a semanticisation of memories over time and with repeated remembering. Nature Communications.

[CR54] Lloyd-Hughes B (2014). The impracticality of a universal drought definition. Theoretical and Applied Climatology.

[CR55] Löwenhielm, M. 2008. Dricksvatten från enskilda vattentäkter [English: Drinking water from private water resources]. Swedish National Board of Health and Welfare.

[CR56] Lumbroso D, Vinet F (2012). Tools to improve the production of emergency plans for floods: Are they being used by the people that need them?. Journal of Contingencies and Crisis Management.

[CR57] Lundkvist, E., and S. Andersson. 2018. Torkans effekt på dricksvatten-försörjningen i Mälarregionen—En studie om kommuners arbete med vattenfrågor utifrån erfarenheter från 2017 [English: Drought effects on drinking water supply in the lake Mälar region—a study on municipalities’ work with water-related issues based on experience from 2017]. Uppsala: Uppsala University.

[CR58] Matti S, Lundmark C, Ek K (2017). Managing participation: Prospects for learning and legitimacy-creation in Swedish water management. Water Policy.

[CR59] Maxe, L. 2021. Vattenkvalitet enskilda brunnar—dataunderlag [English: Water quality of privvate wells - underlying data] (No. 2021:10), SGU-rapport. SGU - Geological Survey of Sweden, Uppsala, Sweden.

[CR60] McKee, T.B., N.J. Doesken, AND J. Kleist. 1993. The relationship of drought frequency and duration to time scales, in: Proceedings of the 8th Conference on Applied Climatology. Presented at the Conference on Applied Climatology, American Meteorological Society Boston, MA, Anaheim, California, USA, pp. 179–183.

[CR61] Measham TG, Preston BL, Smith TF, Brooke C, Gorddard R, Withycombe G, Morrison C (2011). Adapting to climate change through local municipal planning: Barriers and challenges. Mitigation and Adaptation Strategies for Global Change.

[CR63] Nordgren J, Stults M, Meerow S (2016). Supporting local climate change adaptation: Where we are and where we need to go. Environmental Science & Policy.

[CR64] November V, Delaloye R, Penelas M (2007). Crisis management and warning procedures. Journal of Alpine Research | Revue De Géographie Alpine.

[CR65] O’Neill E, Brereton F, Shahumyan H, Clinch JP (2016). The impact of perceived flood exposure on flood-risk perception: The role of distance. Risk Analysis.

[CR66] Pearson K (1920). Notes on the history of correlation. Biometrika.

[CR67] Pecl GT, Araújo MB, Bell JD, Blanchard J, Bonebrake TC, Chen IC, Clark TD, Colwell RK (2017). Biodiversity redistribution under climate change: Impacts on ecosystems and human well-being. Science.

[CR68] Pettersson M, van Rijswick M, Suykens C, Alexander M, Ek K, Priest S (2017). Assessing the legitimacy of flood risk governance arrangements in Europe: Insights from intra-country evaluations. Null.

[CR69] Pfister, C. 2011. “The monster swallows you”: disaster memory and risk culture in western Europe, 1500–2000. RCC perspectives 1–23.

[CR70] Quiring SM (2009). Developing objective operational definitions for monitoring drought. Journal of Applied Meteorology and Climatology.

[CR71] Raikes J, Smith TF, Jacobson C, Baldwin C (2019). Pre-disaster planning and preparedness for floods and droughts: A systematic review. International Journal of Disaster Risk Reduction.

[CR72] Rapp M (2018). Årets torka beräknas till tio miljarder kronor.

[CR73] Ridolfi E, Albrecht F, Di Baldassarre G (2020). Exploring the role of risk perception in influencing flood losses over time. Hydrological Sciences Journal.

[CR74] Rossi G, Cancelliere A, Pereira LS, Oweis T, Shatanawi M (2003). Tools for drought mitigation in mediterranean regions.

[CR75] Salam R, Ghose B, Shill BK, Islam MdA, Islam ARMdT, Sattar MdA, Alam GMM, Ahmed B (2021). Perceived and actual risks of drought: Household and expert views from the lower Teesta River Basin of northern Bangladesh. Natural Hazards.

[CR76] Salvadori G, De Michele C, Durante F (2011). On the return period and design in a multivariate framework. Hydrology and Earth System Sciences.

[CR77] Schlaepfer DR, Bradford JB, Lauenroth WK, Munson SM, Tietjen B, Hall SA, Wilson SD, Duniway MC (2017). Climate change reduces extent of temperate drylands and intensifies drought in deep soils. Nature Communications.

[CR78] Şen Z (2015). Applied drought modeling, prediction, and mitigation.

[CR79] SGU. 2022. Grundvattennivåer [English: Groundwater levels]. https://www.sgu.se/grundvatten/grundvattennivaer/. Accessed 18 Nov 2022.

[CR80] Smakhtin VU, Schipper ELF (2008). Droughts: The impact of semantics and perceptions. Water Policy.

[CR81] SMHI. 2022. Varningar och meddelanden - vattenbrist [English: Warnings and messages—water shortages]. https://api.screen9.com/embed/8lZ5R8oyIhc0yY3arbGLPw. Accessed 18 Nov 2022.

[CR82] Spearman C (1904). The proof and measurement of association between two things. American Journal of Psychology.

[CR83] Statistics Sweden. 2019. Agricultural statistics 2019 including food statistics—tables, agricultural statistics. Swedish Board of Agriculture (SBA), Jönköping, Sweden.

[CR84] Statistics Sweden. 2020. Water use in Sweden 2020 (water withdrawal and water use in Sweden 2022:1 No. 2022 MI27), MI27—water withdrawal and water use in Sweden 2022:1. Statistiska Centralbyrån, Solna, Sweden.

[CR85] Steg L, Sievers I (2000). Cultural theory and individual perceptions of environmental risks. Environment and Behavior.

[CR86] Steinemann A (2003). Drought indicators and triggers: A stochastic approach to evaluation1. JAWRA Journal of the American Water Resources Association.

[CR87] Stensen, K., A. Krundegård, K. Rasmusson, B. Matti, and N. Hjerdt. 2019. Sveriges vattentillgång utifrån perspektivet vattenbrist och torka—Delrapport 1 i regeringsuppdrag om åtgärder för att motverka vattenbrist i ytvattentäkter. [English: Sweden’s water supply from the perspective of water shortages and droughts—sub-report 1 for the government assignment on measures to counteract water shortages in surface water sources. (No. 120), Hydrologi. Swedish Meteorological and Hydrological Institute (SMHI), Norrköping, Sweden.

[CR88] Sveriges Radio. 2018. Dry pastures could force farmers to kill off livestock early - Radio Sweden. https://sverigesradio.se/sida/artikel.aspx?programid=2054&artikel=6977568. Accessed 11 Apr 2021.

[CR89] Swedish Board of Agriculture. 2019. Långsiktiga effekter av torkan 2018 och hur jordbruket kan bli mer motståndskraftigt mot extremväder [English: Long-term effects of the 2018 drought and how farming can become more resilient to extreme weather] (No. 2019a:13). Swedish Board of Agriculture, Jönköping, Sweden. https://www2.jordbruksverket.se/download/18.21625ee16a16bf0cc0eed70/1555396324560/ra19_13.pdf. Accessed 10 Feb 2023.

[CR90] Swedish Defence University. 2019. Förutsättningar för krisberedskap och totalförsvar i Sverige [English: Conditions for crisis preparedness and total defense in Sweden] (No. 930/2011). Swedish Defence University, Stockholm, Sweden.

[CR91] Swedish Government. 2009. Ordinance (SFS 2009:956) on Flood Risk.

[CR92] Swedish Government, 2018a. Torkan och värmen 2018 [English: Drought and Heat 2018]. Regeringskansliet. https://www.regeringen.se/regeringens-politik/torkan-och-varmen-2018b/. Accessed 26 Nov 2018.

[CR93] Swedish Government. 2018b. Nationell strategi för klimatanpassning (Prop. 2017/18:163) [English: National Strategy for climate change adaptation, Gov. Bill 2017/18:163].

[CR94] Teutschbein C, Quesada Montano B, Todorović A, Grabs T (2022). Streamflow droughts in Sweden: Spatiotemporal patterns emerging from six decades of observations. Journal of Hydrology: Regional Studies.

[CR520] Teutschbein, C., E. Jonsson, A. Todorović, F. Tootoonchi, E. Stenfors, and T. Grabs. 2022b. Future Drought Propagation through the Water-Energy-Food-Ecosystem Nexus – a Nordic Perspective. *Journal of Hydrology* 128963. 10.1016/j.jhydrol.2022.128963.

[CR95] The Local. 2018. What you need to know about Sweden’s historic wildfire outbreak—the local. https://www.thelocal.se/20180717/sweden-battles-most-serious-wildfire-situation-of-modern-times-heres-what-you-need-to-know. Accessed 26 Nov 2018.

[CR96] Tootoonchi F, Sadegh M, Haerter JO, Räty O, Grabs T, Teutschbein C (2022). Copulas for hydroclimatic analysis: A practice-oriented overview. Wires Water.

[CR97] Tsakiris G, Nalbantis I, Vangelis H, Verbeiren B, Huysmans M, Tychon B, Jacquemin I, Canters F (2013). A system-based paradigm of drought analysis for operational management. Water Resources Management.

[CR98] TT Nyhetsbyrån. 2018. Översvämningar kostar miljontals kronor—oklart vem som ska betala [English: Floods cost millions of Swedish kronas - unclear who should pay]. Ny Teknik. https://www.nyteknik.se/nyheter/oversvamningar-kostar-miljontals-kronor-oklart-vem-som-ska-betala/875052. Accessed 23 Mar 2023.

[CR99] Tversky A, Kahneman D (1973). Availability: A heuristic for judging frequency and probability. Cognitive Psychology.

[CR100] UNDRR. 2019. Global Assessment Report on Disaster Risk Reduction 2019. United Nations Office for Disaster Risk Reduction (UNDRR), Geneva, Switzerland.

[CR101] UNDRR. 2021. GAR Special report on drought 2021, Global assessment report on disaster risk reduction. United Nations Office for Disaster Risk Reduction, Geneva, Switzerland.

[CR102] Van Loon AF (2015). Hydrological drought explained. WIREs. Water.

[CR103] Van Loon AF, Van Lanen HAJ (2012). A process-based typology of hydrological drought. Hydrology and Earth System Sciences.

[CR104] Vattenmyndigheterna. 2018. Vattenbristuppdragen—hur rustar vi oss mot nästa torka? [English: Water shortage mission - how do we prepare for the next drought?] http://www.vattenmyndigheterna.se:80/Sv/nyheter/2018/Sidor/Vattenbristuppdragen%E2%80%93hur-rustar-vi-oss-mot-nasta-torka-.aspx. Accessed 26 Nov 2018.

[CR105] Veijalainen N, Ahopelto L, Marttunen M, Jääskeläinen J, Britschgi R, Orvomaa M, Belinskij A, Keskinen M (2019). Severe drought in Finland: Modeling effects on water resources and assessing climate change impacts. Sustainability.

[CR106] Vicente P, Reis E (2010). Using questionnaire design to fight nonresponse bias in web surveys. Social Science Computer Review.

[CR107] Viglione A, Di Baldassarre G, Brandimarte L, Kuil L, Carr G, Salinas JL, Scolobig A, Blöschl G (2014). Insights from socio-hydrology modelling on dealing with flood risk—roles of collective memory, risk-taking attitude and trust. Journal of Hydrology, Creating Partnerships between Hydrology and Social Science: A Priority for Progress.

[CR108] Vikström, K. 2018. Så mycket kan skogsbränderna kosta [English: How much the forest fires could cost]. SVT nyheter. https://www.svt.se/nyheter/lokalt/norrbotten/sa-mycket-kan-skogsbranderna-kosta. Accessed 23 Mar 2023.

[CR109] Vogt, J.V., G. Naumann, D. Masante, J. Spinoni, C. Cammalleri, W. Erian, F. Pischke, R. Pulwarty, et al. 2018. Drought risk assessment and management (No. EUR 29464 EN), JRC technical reports. Publications Office of the European Union, Luxembourg.

[CR110] Wilhite D (1996). A methodology for drought preparedness. Natural Hazards.

[CR111] Wilhite DA (2019). Integrated drought management: Moving from managing disasters to managing risk in the Mediterranean region. Euro-Mediterranean Journal for Environmental Integration.

[CR112] Wilhite DA, Hayes MJ, Knutson C, Smith KH (2000). Planning for drought: Moving from crisis to risk management. JAWRA Journal of the American Water Resources Association.

[CR113] WMO, and GWP. 2016. Handbook of drought indicators and indices, integrated drought management tools and guidelines series. integrated drought management programme (IDMP), Geneva, Switzerland. 10.1201/9781315265551-12

[CR114] Zaidman MD, Rees HG, Young AR (2002). Spatio-temporal development of streamflow droughts in north-west Europe. Hydrology and Earth System Sciences.

