# Supplementary material for: Drought hazards and stakeholder perception: Unraveling the interlinkages between drought severity, perceived impacts, preparedness, and management
Source: Ambio. 2023 Apr 3;52(7):1262–81. doi: 10.1007/s13280-023-01849-w (PMC10247940; doi:10.1007/s13280-023-01849-w)
Supplement: Supplementary file 1 — Supplementary file1 (PDF 733 KB) [file 13280_2023_1849_MOESM1_ESM.pdf]

Ambio

Electronic Supplementary Material

This supplementary information has not been peer reviewed.

Drought hazards and stakeholder perception: Unraveling the interlinkages between drought severity, perceived impacts, preparedness and management

Claudia Teutschbein, Frederike Albrecht, Malgorzata Blicharska, Faranak Tootoonchi, Elin Stenfors and Thomas Grabs

S1. Study area Sweden

The study was conducted in Sweden (Fig. S 1), a country in northern Europe, which covers an area of approximately 408,000 km^2. Sweden is subdivided into 290 municipalities (Fig. S 1a) organized within 21 counties (Gunningberg et al., 2012). While the counties are the top-level administrative and political subdivisions, municipalities are the local government bodies (Gunningberg et al. 2012). Sweden has a total population of 10.3 million (SCB 2020), thus featuring an average population density of 25.3 inhabitants per square kilometer, with denser populated areas in the south and along the coastline, and less populated areas in the northern inland areas (Fig. S 1a).

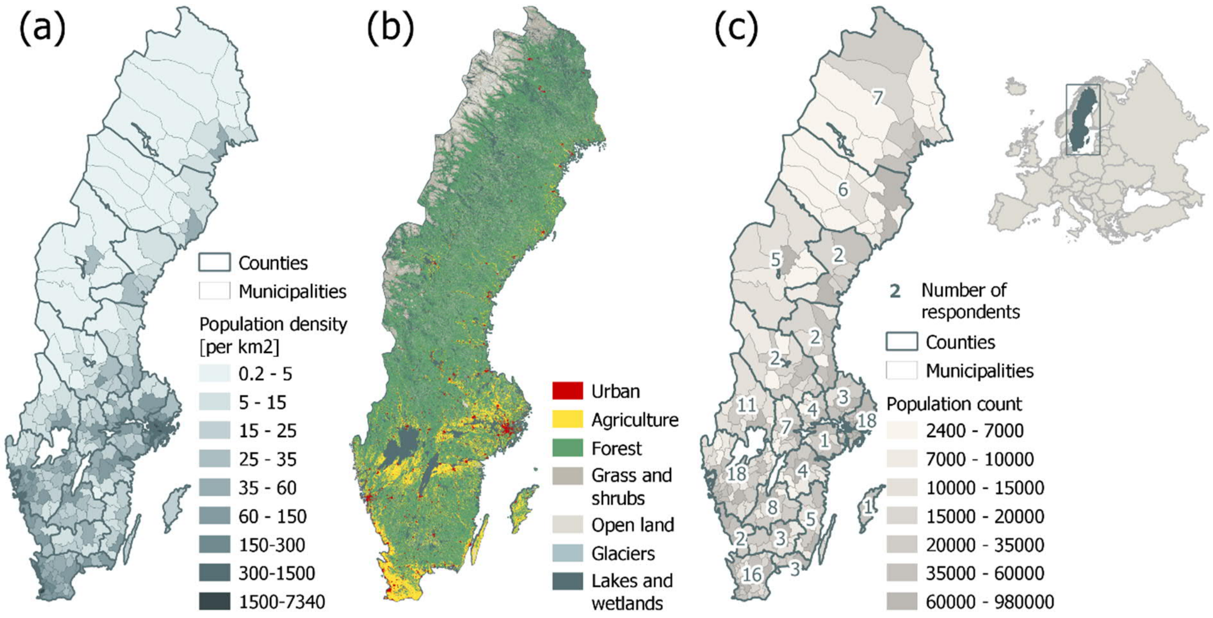

Fig. S 1: Map of Sweden's (a) administrative boundaries (counties and municipalities) and population density, (b) main land cover types, and (c) population count and number of survey respondents.

More than two thirds (69%) of Sweden's area are currently covered by forests (SLU 2015), 9% by wetlands, 8% by shrubs and grass land, 8% by agriculture (mostly in southern Sweden), 3% by human settlements and the remaining 3% are open land (Fig. S 1b). Although Sweden had an average annual precipitation of only 624 mm during the period 2013-2017 and, thus, received much less water than the global average (30th percentile, cf. FAO, 2020a), it has historically been known for its abundant water resources. This is because less than 50% of the precipitation is lost by evapotranspiration, leaving a large portion of water in the landscape to recharge groundwater resources before the water eventually flushes into the Baltic Sea, the Kattegat or Skagerrak. Based on freshwater data provided by FAO (2020b), Sweden thus lies on the 75th percentile in terms of annually renewable freshwater resources (i.e., water availability from precipitation, groundwater recharge and surface inflows), clearly indicating that it is - under normal conditions - one of the 50 water-richest countries in the world.

S2. Water management in Sweden

Water-related questions are governed by policies at different governance levels, from global Sustainable Development Goals (SDGs) (Ait-Kadi 2016), through the European Water Framework Directive (WFD) 2000/60/EC (Directive 2000/60/EC 2000) to national level Environmental Code (Swedish Government 1998) and Environmental Quality Objectives (EQOs). Governance of hydrological risk (both drought & flood) in Sweden is not linked to a distinct policy domain. Actors, rules, resources and discourses pertaining to droughts and drought risk are spread across several policy areas, including e.g. spatial planning, water management/planning, environmental protection and emergency management (Ek et al. 2016). They also encompass governance levels from national to local: As a result of the 2004 translation of the WFD into Swedish law, Sweden has been divided into five water districts that are governed by river basin district authorities

(RBDAs) that coordinate the efforts to implement the WFD at regional level. The RBDAs ratify the river basin management plans and have decision-making authority (Sevä and Jagers 2013). Each river basin district contains several County Administrative Boards (CABs), which are supervised by the RBDAs. These CABs coordinate the work in the counties, ensure that laws and regulations set by the Swedish government are implemented at the regional and local levels, and provide policy recommendations to the RBDAs.

While both the RBDAs and CABs play a central role in protecting and improving water quality and quantity, the water governance system largely relies on the municipal self-government, which is important in development of drought management strategies and has legal obligation to create local action plans. The Swedish Civil Contingencies Agency (MSB) offers support and guidance for municipalities to fulfil this task.

Municipalities generally have a strong role in decision-making processes in Sweden, which is a direct consequence of the strong decentralization (Lidström 2020). However, this decentralization has also been criticized to limit the involvement of all relevant stakeholders in risk management (Johannessen et al. 2019). Nonetheless, municipalities act as operators who are directly responsible for local water management and the implementation of necessary measures to protect local surface and groundwater bodies that are used (or maybe used in the future) for drinking water supply. It should be stressed that Sweden has implemented the principle of responsibility/accountability (‘Ansvarsprincipen’), which implies that those responsible for water management in ordinary times will also be responsible in times of crisis, such as water shortages (Swedish Defence University 2019). The responsibilities of municipalities also include a large part of all public services and infrastructure planning, such as drinking water distribution and sewerage systems (Carlsson-Kanyama 2013). To ensure a long-term protection of local water resources, municipalities are obliged to introduce and maintain water protection areas, and are required to design and monitor local water supply plans. This is an essential prerequisite for achieving both the WFD and the EQOs (Swedish River Basin District Authorities 2016).

S3. Survey design

The survey included background questions about local water resources, perception of future risk for floods and droughts, the current work with water issues, including the presence of action plans for floods and droughts, and need for research and collaboration. The core of the survey consisted of a set of questions related to the municipalities’ perception of the severity and impacts of the 2018 drought, directly followed by the same set of questions for the 2017 drought, including an assessment of the municipalities’ drought preparedness and management of these events (Fig. S 2).

Questions were kept short with 30 (partly nested) groups of questions (Fig. S 2), phrased clearly and structured to avoid unnecessary biases and to maximize the information content provided by respondent replies. The use of rating-scale questions with regards to drought severity, impact, preparedness and management was implemented to allow systematic statistical analysis of the outcomes.

The survey originally received 127 unique responses with completion rates ranging from 13% to 100% (average 79%). After an initial screening, responses that solely included answers to the background questions (without replying to any of the other five key sections) were removed. In case of multiple responses from different people within one municipality, we kept only the one response with a highest completion rate. Out of the 290 municipalities, this resulted in 118 unique responses from different municipalities (response rate ca. 41 %) with completion rates ranging from 30% to 100% (average 89%). These responses covered all 21 counties, with generally more respondents from the highly-populated areas surrounding the three largest Swedish cities, Stockholm, Gothenburg and Malmö. Most of the respondents (52%) answered in their role as head of department, including for example the municipalities’ departments for drinking & waste water, environment, sanitation or urban planning & infrastructure. This was followed by operators/technicians/engineers, which were the second largest group to respond (19%). Other respondents included for instance strategists (6%), managers/supervisors (6%), operational managers (5%), and coordinators (3%).

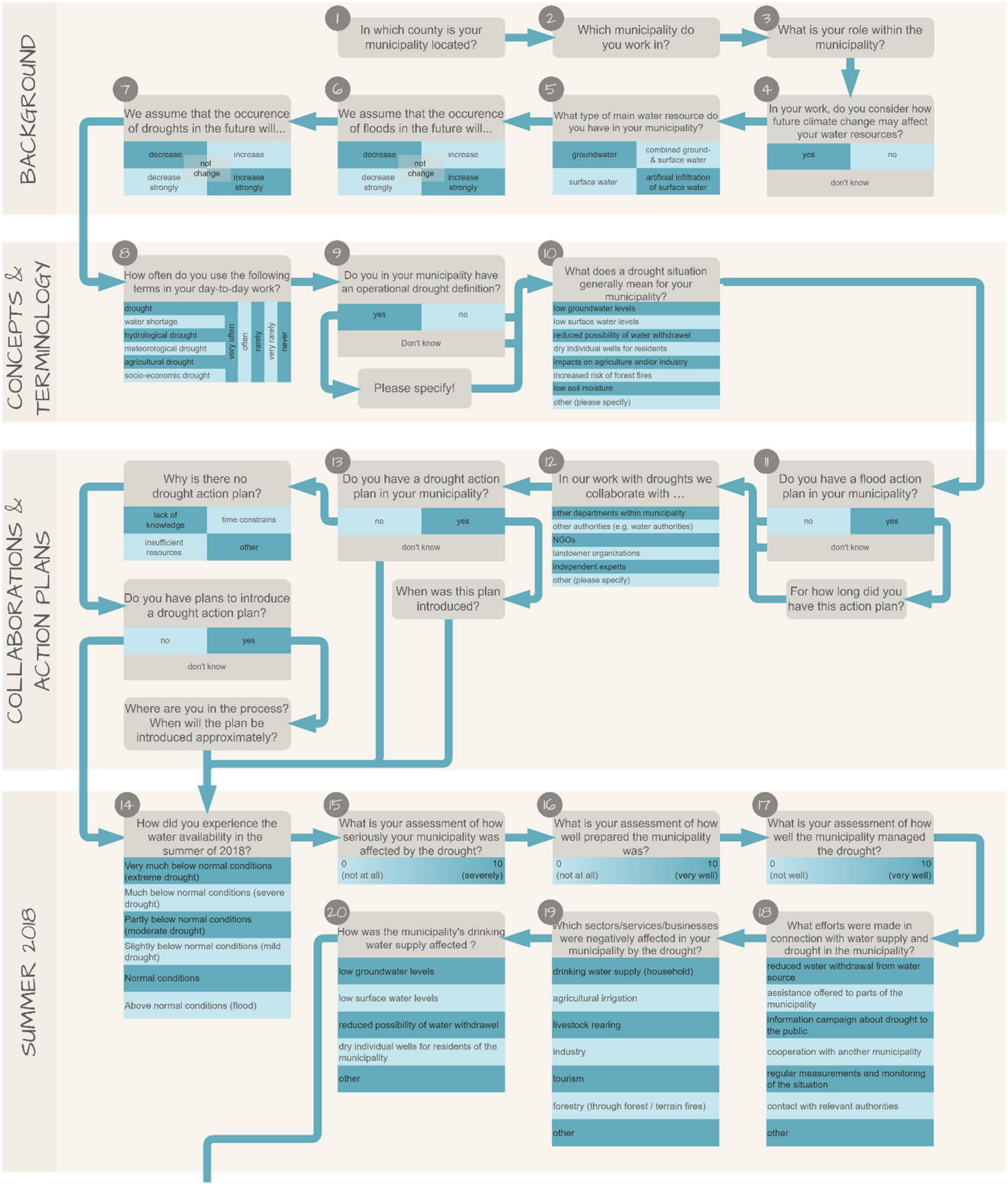

Fig. S 2: Design of the online survey (continues on next page).

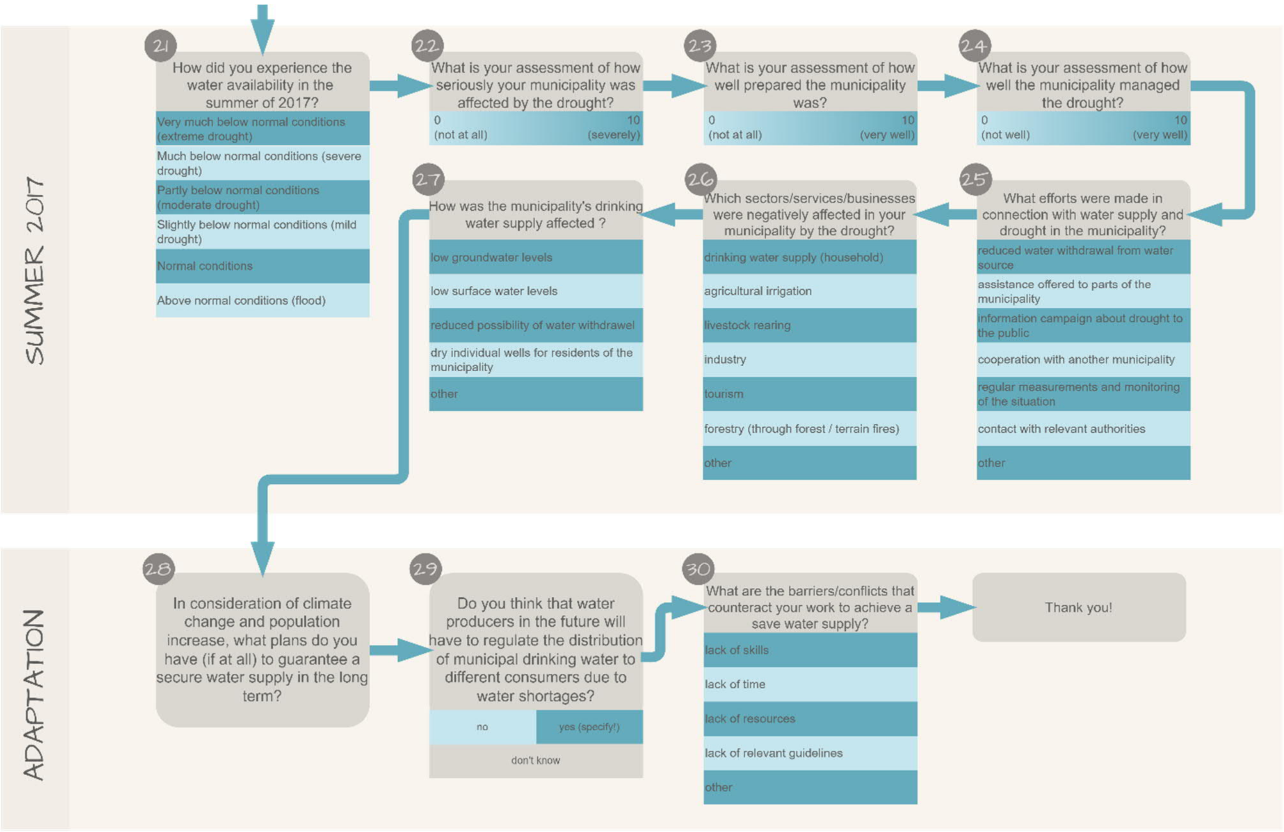

Fig. S 2 continued.

S4. Hydroclimatic Data and Analysis Methods

S4.1. Precipitation deficit

Gridded daily mean values of precipitation and temperature were downloaded from the publicly accessible PTHBV database (SMHI 2005), which provides a spatially interpolated 4 km x 4 km national grid (Johansson 2002) hosted by the Swedish Meteorological and Hydrological Institute (SMHI). For each municipality, average monthly precipitation and temperature were computed for the period 1961-2020. The precipitation data was then analyzed using the Standardized Precipitation Index (SPI) originally developed by McKee et al. (1993). The SPI provides a dimensionless anomaly from normal situations. It is computed by first fitting a probability distribution function to the entire time series of monthly mean precipitation (1961-2020), which is then transformed into a normal distribution with zero mean and a standard deviation of one (so-called z-scores). The SPI then equals these z-scores, which implies that they represent the standard deviation (or anomaly) from the mean. Positive SPI values indicate conditions above normal (i.e. wet conditions), while negative values represent below-normal conditions (i.e. drought conditions).

S4.2. Groundwater deficit

From a set of 1193 groundwater wells monitored by the Swedish Geological Survey (SGU), we downloaded a sub-set of 181 wells that had measurements available for the period 1981-2018 from SGU's public webpage and that had during this time less than 10% of missing values. After calculating monthly mean values for the period 1981-2018 for each of the stations, we computed the station-wise Standardized Groundwater Index (SGI), which was developed by Bloomfield and Marchant (2013) and is based on the same procedure as the SPI explained above. The station-wise SGI values where then interpolated to cover entire Sweden by using Inverse Distance Weighting (Lu and Wong 2008) and thereafter averaged for each municipality.

Both the SPI and the SGI can be calculated at various time scales (e.g. 1, 3, 6, 12, 24 months etc.), while we here chose to analyze the 6-month SPI and SGI (written SPI6 and SGI6, respectively) for the month of August for 2017 and 2018, which integrates precipitation respective groundwater anomalies during the spring and warm

summer months from March to August and is appropriate to reflect both the agricultural and hydrological (i.e., surface and groundwater) drought conditions. The severity of the precipitation and groundwater deficits was assessed with a classification scheme following closely the categories provided by Lloyd-Hughes and Saunders (2002) ranging from normal conditions through mild, moderate and severe, to extreme droughts (for details see Table S 1). Finally, based on the computed SPI6 and SGI6 values, the spatial extend and severity of the 2017 and 2018 drought were mapped and compared to the perceived severity obtained from the online survey.

Table S 1: Classification of drought conditions by SPI and SGI values and corresponding probabilities, following the scheme of categories by Lloyd-Hughed and Saunders (2002) for precipitation anomalies.

SPI/SGI range	Drought class	Probability [%]
≥ -0.50	normal/near-normal conditions, i.e., no precipitation/ groundwater drought	69.1
-0.99 to -0.50	mild precipitation/groundwater drought	15.0
-1.49 to -1.00	moderate precipitation/groundwater drought	9.2
-1.99 to -1.50	severe precipitation/groundwater drought	4.4
≤ -2.00	extreme precipitation/groundwater drought	2.3

S4.3. Concurrence of precipitation deficit and extreme temperatures

In addition, we also evaluated the 2017 and 2018 droughts based on their empirical return periods. The concept of return periods is commonly applied in hydrological risk assessment (Salvadori et al. 2011) and environmental engineering (Hosking and Wallis 1993) to identify critical events (e.g. floods or droughts) and provide critical information for practitioners. Salvadori et al. (2011) defined return periods as the average time passing between two successive events of a pre-defined magnitude and, thus, it is a statistical notion based on historical data. A 100-year event, for instance, has an exceedance probability of 1% (i.e., 1/100) in any given year.

We allocated empirical return periods to observed annual March-August (spring and summer) precipitation deficits and annual summer temperature anomalies by ordering and ranking them from the most extreme (rank m=1) to the least extreme (rank m=n, where n=60 years), and then used the Weibull formulae (Weibull 1939) to compute the exceedance probabilities ($P = \frac{m}{n+1}$) and return periods ($T = \frac{1}{P} = \frac{n+1}{m}$).

Return periods are traditionally estimated for univariate cases and, therefore, typically rely on one variable such as either precipitation, streamflow or temperature separately. However, as droughts can be driven by both precipitation deficits and/or high temperatures (the latter as a proxy for high evapotranspiration losses), we here focused on the combined statistics of concurrence of precipitation and temperature anomalies (i.e., deviation from long term mean) to better reflect the combined risk. Consequently, we used a bivariate copula approach (Salvadori et al. 2011; AghaKouchak et al. 2015; Tootoonchi et al. 2022) to assess the dependence between annual March-August precipitation deficits and annual summer temperature anomalies (averaged over the months June, July and August) over the period 1961-2020 and to extract their joint return period (Salvadori et al. 2007). After an initial testing of different copula families (including t-copula, Gaussian, Clayton, Frank and Gumbel), the Gaussian survival copula was selected as it best fitted the data and as it is commonly capable of modeling both positive and negative dependences, while benefitting from having fewer parameters.

S5. References

AghaKouchak, A., L. Cheng, O. Mazdiyasni, and A. Farahmand. 2015. Global warming and changes in risk of concurrent climate extremes: Insights from the 2014 California drought. *Geophysical Research Letters*: 8847–8852. doi:10.1002/2014GL062308@10.1002/(ISSN)1944-8007.CALDROUGHT1.

Ait-Kadi, M. 2016. Water for Development and Development for Water: Realizing the Sustainable Development Goals (SDGs) Vision. *Aquatic Procedia* 6: 106–110. doi:10/ggw56j.

Bloomfield, J. P., and B. P. Marchant. 2013. Analysis of groundwater drought building on the standardised precipitation index approach. *Hydrology and Earth System Sciences* 17: 4769–4787. doi:10/f5m79s.

Carlsson-Kanyama, A. 2013. Barriers in municipal climate change adaptation: Results from case studies using backcasting. *Futures*: 13. doi:10.1016/j.futures.2013.02.008.

Directive 2000/60/EC. 2000. *Directive 2000/60/EC of the European Parliament and of the Council of 23 October 2000 establishing a framework for Community action in the field of water policy*.

Ek, K., S. Goytia, M. Pettersson, and E. Spegel. 2016. *Analysing and evaluating flood risk governance in Sweden - Adaptation to climate change?* Utrecht, The Netherlands: STAR - FLOOD Consortium.

FAO. 2020a. Long-term average annual precipitation in depth (mm/year). *AQUASTAT database Database*.

FAO. 2020b. Total renewable water resources (10^9 m3/year). *AQUASTAT database Database*.

Gunningberg, L., A. Hommel, C. Bååth, and E. Idvall. 2012. The first national pressure ulcer prevalence survey in county council and municipality settings in Sweden: National pressure ulcer prevalence survey. *Journal of Evaluation in Clinical Practice*: no-no. doi:10.1111/j.1365-2753.2012.01865.x.

Hosking, J. R. M., and J. R. Wallis. 1993. Some statistics useful in regional frequency analysis. *Water Resources Research* 29: 271–281. doi:10/df96h6.

Johannessen, Å., Å. G. Swartling, C. Wamsler, K. Andersson, J. T. Arran, D. I. H. Vivas, and T. A. Stenström. 2019. Transforming urban water governance through social (triple-loop) learning. *Environmental Policy and Governance* 29: 144–154. doi:10/gfs7c8.

Johansson, B. 2002. Estimation of areal precipitation for hydrological modelling in Sweden. Doctoral dissertation, Report A76, Göteborg, Sweden: Earth Science Centre, Göteborg University.

Lidström, A. 2020. Subnational Sweden, the national state and the EU. *Regional & Federal Studies* 30. Routledge: 137–154. doi:10/ggw6d5.

Lloyd-Hughes, B., and M. A. Saunders. 2002. A drought climatology for Europe. *International Journal of Climatology* 22: 1571–1592. doi:10.1002/joc.846.

Lu, G. Y., and D. W. Wong. 2008. An adaptive inverse-distance weighting spatial interpolation technique. *Computers & Geosciences* 34: 1044–1055. doi:10/d3vxfv.

McKee, T. B., N. J. Doesken, and J. Kleist. 1993. The relationship of drought frequency and duration to time scales. In *Proceedings of the 8th Conference on Applied Climatology*, 17:179–183. Anaheim, California, USA: American Meteorological Society Boston, MA.

Salvadori, G., C. D. Michele, N. T. Kottegoda, and R. Rosso. 2007. Bivariate Analysis Via Copulas. In *Extremes in Nature: An Approach Using Copulas*, 131–175. Dordrecht: Springer Netherlands. doi:10.1007/1-4020-4415-1_3.

Salvadori, G., C. De Michele, and F. Durante. 2011. On the return period and design in a multivariate framework. *Hydrology and Earth System Sciences* 15: 3293–3305. doi:10/d2zdsm.

SCB. 2020. Befolkningsstatistik [en: Population statistics]. *Statistiska Centralbyrån*.

Sevä, M., and S. C. Jagers. 2013. Inspecting environmental management from within: The role of street-level bureaucrats in environmental policy implementation. *Journal of Environmental Management* 128: 1060–1070. doi:10.1016/j.jenvman.2013.06.038.

SLU. 2015. *Forest statistics 2015*. Official Statistics of Sweden. Umeå, Sweden: Swedish University of Agricultural Sciences (SLU).

SMHI. 2005. *PTHBV klimatdatabas för hydrologiska beräkningar [PTHBV Climate Database for Hydrological Calculations]*. Produktblad. Norrköping, Sweden: Swedish Meteorological and Hydrological Institute (SMHI).

Swedish Defence University. 2019. *Förutsättningar för krisberedskap och totalförsvar i Sverige [en: Conditions for crisis preparedness and total defense in Sweden]*. 930/2011. Stockholm, Sweden: Swedish Defence University.

Swedish Government. 1998. The Swedish Environmental Code (SFS 1998:808).

Swedish River Basin District Authorities. 2016. *Förvaltningsplan Södra Östersjöns vattendistrikt 2016–2021. Del 5, Vattenförvaltning 2016–2021 - Strategiska val inom vattenförvaltning kommande år [Management plan Souther Baltic Broper 2016-2021. Part 5, Water Management 2016-2021 - Strategic choices within water management in coming years]*. Kalmar, Sweden: County Administrative Board Kalmar.

Tootoonchi, F., M. Sadegh, J. O. Haerter, O. Räty, T. Grabs, and C. Teutschbein. 2022. Copulas for hydroclimatic analysis: A practice-oriented overview. *WIREs Water* 9: e1579. doi:10.1002/wat2.1579.

Weibull, W. 1939. *A statistical theory of the strength of materials*. 151. Ingeniörsvetenskapsakademiens Handlingar. Stockholm, Sweden: Generalstabens Litografiska Anstalts Förlag.
